# Looking for compensation at multiple scales in a wetland bird community

**DOI:** 10.1002/ece3.8876

**Published:** 2022-06-02

**Authors:** Frédéric Barraquand, Coralie Picoche, Christelle Aluome, Laure Carassou, Claude Feigné

**Affiliations:** ^1^ Institute of Mathematics of Bordeaux University of Bordeaux and CNRS Talence France; ^2^ 84269 Integrative and Theoretical Ecology LabEx COTE University of Bordeaux Pessac France; ^3^ ISPA Bordeaux Sciences Agro & INRAE Villenave d'Ornon France; ^4^ EABX INRAE Cestas France; ^5^ Teich Ornithological Reserve PNR Landes Gascogne Le Teich France

**Keywords:** biodiversity, birds, compensation, synchrony, time series, wavelets

## Abstract

Compensatory dynamics, during which community composition shifts despite a near‐constant total community size, are usually rare: Synchronous dynamics prevail in natural communities. This is a puzzle for ecologists, because of the key role of compensation in explaining the relation between biodiversity and ecosystem functioning. However, most studies so far have considered compensation in either plants or planktonic organisms, so that evidence for the generality of such synchrony is limited. Here, we extend analyses of community‐level synchrony to wetland birds. We analyze a 35‐year monthly survey of a community where we suspected that compensation might occur due to potential competition and changes in water levels, favoring birds with different habitat preferences. We perform both year‐to‐year analyses by season, using a compensation/synchrony index, and multiscale analyses using a wavelet‐based measure, which allows for both scale‐ and time‐dependence. We analyze synchrony both within and between guilds, with guilds defined either as tightknit phylogenetic groups or as larger functional groups. We find that abundance and biomass compensation are rare, likely due to the synchronizing influence of climate (and other drivers) on birds, even after considering several temporal scales of covariation (during either cold or warm seasons, above or below the annual scale). Negative covariation in abundance at the guild or community level did only appear at the scale of a few months or several years. We also found that synchrony varies with taxonomic and functional scale: The rare cases where compensation appeared consistently in year‐to‐year analyses were *between* rather than *within* functional groups. Our results suggest that abundance compensation may have more potential to emerge between broad functional groups rather than between species, and at relatively long temporal scales (multiple years for vertebrates), above that of the dominant synchronizing driver.

## Graphical abstract

While abundance compensation is key to explain the increased stability of communities relative to populations, it is rarely found in field data. Using a long‐term wetland bird community survey, we show that while synchrony dominates at most scales, compensation can be found between rather than within functional groups in the winter season, and at long temporal scales. This suggests that zooming out functionally and temporally may help in finding compensation in community time series.

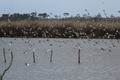

## 1 INTRODUCTION

Density compensation occurs when individuals of a given species replace individuals of other species within a community, either because of explicit competitive processes or because of shifts in environmental drivers that change selection pressures (Gonzalez & Loreau, 2009). The community as a whole then exhibits lower abundance variation than its constituent species (Gross et al., 2014): Some degree of compensation or asynchrony is therefore a prerequisite to stabilization at the community level (Loreau & de Mazancourt, 2013).

Understanding why environmental variation may lead to compensation is relatively easy: If species have different environmental preferences (e.g., thermal optima), and the environment changes overtime, different species will be fittest at different points in time. As a consequence, relative abundances will shift overtime even though the community abundance or biomass as a whole may remain relatively stable (Gonzalez & Loreau, 2009). However, the conditions for compensation to happen also depend on the particulars of the interactions between and within species in the community.

Compensation is particularly likely to occur when temporal environmental variation combines with a space constraint or with a strongly limiting resource, so that individuals are close to competing in a zero‐sum game (sensu Hubbell, 2001 or lottery‐style models, Chesson, 1994). When the total community size is constant overtime, and the composition fluctuates, negative covariation between abundances then emerges by design (Loreau & de Mazancourt, 2008) as no species can increase without at least another species decreasing in abundance. Outside of this zero‐sum scenario, in models where Lotka‐Volterra competition is combined with temporal environmental variability, theoretical research has revealed that increased interspecific competition might not always increase species compensation (Ives et al., 1999) and might even decrease it (i.e., increase species synchrony instead, Loreau & de Mazancourt, 2008, 2013), although this depends on the fluctuation regime. Thus, in a world where total community size varies, predicting whether compensatory dynamics can occur is intrinsically difficult (van Klink et al., 2019).

Early investigations of the frequency of synchronous vs compensatory dynamics focused on the variance ratio, that is, the variance of the community biomass divided by the sum of the variances of the component species biomasses (Gonzalez & Loreau, 2009; Houlahan et al., 2007). Unfortunately, this metric is not appropriate for communities subjected to community‐wide environmental forcing (Ranta et al., 2008), because a main environmental driver (e.g., temperature or light) may synchronize species abundances or growth rates at some temporal scale, creating large variance in community‐wide biomass, in spite of strongly competitive dynamics. Further research has therefore focused on specific timeframes during which compensatory dynamics may be found (e.g., below the annual scale at which temperature fluctuations tend to synchronize species dynamics, Vasseur et al., 2014).

Despite efforts to look for more meaningful temporal scales in community‐level time series, temporal compensation has remained surprisingly elusive in the field (Houlahan et al., 2007; Vasseur et al., 2014); but see Christensen et al. (2018) and Morgan Ernest et al. (2008). Most datasets used so far to evaluate temporal compensation vs synchrony involve planktonic organisms (Vasseur et al., 2014; Vasseur & Gaedke, 2007) or terrestrial plants (Bai et al., 2004; Gross et al., 2014; Houlahan et al., 2007; though see Bell et al., 2014 in fishes, Morgan Ernest et al., 2008 in mammals and van Klink et al., 2019 in beetles). Here, we take advantage of a long‐term bird abundance time series in a natural reserve, with records every month for 35 years, allowing us to dig deeper into patterns of synchrony, at several temporal and taxonomic or functional scales.

The taxonomic or functional scale considered should indeed be a main modulator of synchrony/compensation. On the one hand, compensation can be high between morphologically similar and closely related species. If two species of ducks A and B share almost the same niche, individuals from either species experience similar competition from species A or B, and should feel the effects of individuals of other species in the community identically. This favors priority effects (Fukami, 2015), with chance due to movement events determining whether species A or B locally dominates, which can then provide compensation at the landscape level (Loreau et al., 2003). On the other hand, it could be argued that these two similar duck species will precisely respond in similar ways to environmental variables, which tends to obfuscate compensation. Hence, more dissimilar species or groups (within the same trophic level nonetheless) could exhibit more compensation (Bai et al., 2004; Morin et al., 2014; van Klink et al., 2019) because they are more likely to respond to the environment in an asynchronous manner (*sensu* Loreau & de Mazancourt, 2013). Surprisingly, such compensation *between* guilds has been less well explored empirically than *within* guilds, even though there is actually some empirical evidence for compensation between dissimilar guilds (Bai et al., 2004; Roscher et al., 2011; Sinclair et al., 2013; van Klink et al., 2019). In this paper, we explore the level of compensation/synchrony within or between guilds of a wetland bird community, along either taxonomic or functional classifications. Although a functional classification might appear intuitively more appealing, our knowledge of functional traits is necessarily partial and imperfect, so that a taxonomic description can sometimes be preferable (Clark, 2016). Our dataset is ideally suited to examine the presence of synchrony or compensation at different scales given that (i) it is a highly temporally resolved time series with respect to the species typical generation times, but it also extends well beyond generation time (timespan of 35 years) and (ii) the reserve where the data have been collected was subjected to a major management change c. 2006 (change in water levels), favoring different types of wetland birds (so that over long timescales, there is a real potential for changes in community composition).

## 2 MATERIAL AND METHODS

### 2.1 Data

**FIGURE 1:**
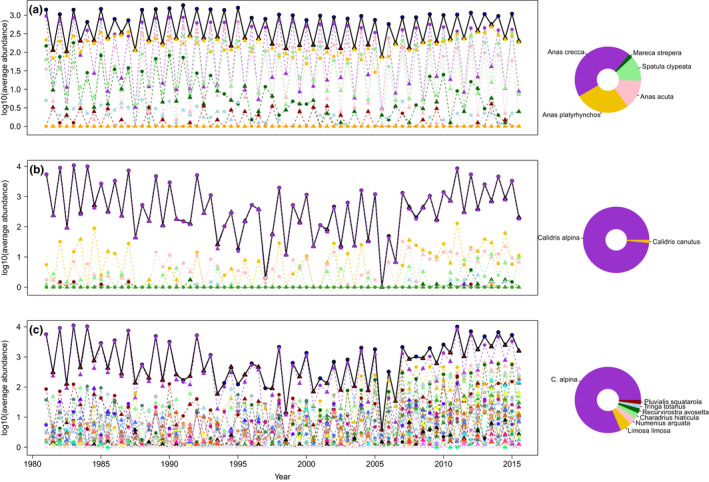
Time series of seasonally averaged abundance for ducks of the tribe *Anatini* (a), calidrids (b, *Calidris* genus), and all waders (c, including calidrids). The solid black lines (on top of each panel) represent the summed average abundances for each guild, dotted lines represent average abundance for each species. Circles represent the cold season and triangles, the warm season. The colored symbols below the curves represent each species abundances, with species composition on the right side on the donut plots for the most abundant species (over 1% of relative abundance in the group considered). We added 1 to abundances before log‐transforming to avoid issues with zero values

The monthly time series used for the statistical analyses have been collected at the Teich Ornithological Reserve, Arcachon Bay, France (44.64°N/−1.02°E), by the staff of the Teich reserve, over the whole study period (1981–2016). A species list of the frequent birds is provided in Appendix S1. The reserve comprises 120 ha of wetlands, and the counts have been aggregated at the reserve scale (summed over 18 sectors where the counts are actually performed, using binoculars). We use for each species the maximum observed abundance over a month, which provides a “monthly snapshot” of bird abundance, that has been used to monitor the reserve since its inception. When abundance values are not reported for certain species and months, we replace them by zeroes. Given the sustained observation effort (all sectors are patrolled multiple times throughout the month by the staff and the reserve is visited every day by birdwatchers who communicate their findings to the reserve staff), we consider that the absence of counts for a given species signals its true absence from the reserve. This creates some zero abundances for rare species at the monthly scale. We have not attempted to “correct” those zeroes (e.g., inferring the “missing” data with a model assuming that our reserve is a subsample of a regional population) because doing so would have compromised the patterns of local synchrony/compensation. However, we did check that having such zeroes in the monthly time series cannot affect our conclusions (see Appendix S6). In the statistical analyses, we use seasonally averaged abundances (plotted in Figure 1), and the original monthly data (presented in Appendix S2). We defined two seasons based on observations of bird presence. We defined a “warm season,” from May to August, and a “cold season” as the months between November and February of the following year. From an ecological viewpoint, this seasonal classification separates wintering birds from spring and summer residents (some of whom are breeding). This makes sense biologically because the two communities have different requirements and could respond differentially to abiotic drivers. It is also useful from a more statistical perspective, as there is a partial shift in composition between the seasons, even though winter and summer communities greatly overlap (i.e., abundant species in winter can also have substantial albeit smaller abundances in summer—although summer residents may be different individuals). The dynamics of species abundances in the Teich reserve bird community show a marked signature of seasonality (Figure 1).

### 2.2 Bird taxonomic and functional groups

The reserve is dominated by waders and waterfowl (ducks, geese, and swans). These two functional groups collectively represent 68% of the total number of observed birds over the years and are always present on site. Two fairly common phylogenetic groups, both in abundance and in occurrence, are members of the *Anatini* tribe (corresponding previously to the *Anas* genus, Gonzalez et al., 2009) in ducks and members of the *Calidris* genus in waders. Waders and ducks have different environmental preferences, with ducks (and waterfowl more generally) preferring water levels allowing them to dabble (or dive for *Aythini*), while waders usually forage on mudflats. A list of all birds found frequently in the reserve is presented in Appendix S1; aside from waders and waterfowl, other common species include herons, egrets, and cormorants (see below). Among the fish eaters, grebes and gulls were frequently counted; a few raptors were present as well.

To examine compensation *between* and *within* the wader and waterfowl categories, we contrasted analyses using a taxonomic classification of the species (i.e., between and within phylogenetic groups such as genera) and a functional classification of the species (26 species of waders vs 17 species of waterfowl). The waterfowl group includes all anatids (ducks, geese, and swans in particular) and the common coot *(Fulica atra*, an abundant species here, which is a Rallidae but resembles a duck in morphology and foraging habits; hence its inclusion).

In addition to our main analyses on waders and waterfowl, we also focused on a set of species that were known to exhibit potentially compensatory dynamics through competition for roosting sites: the great cormorant *(Phalacrocorax carbo)*, the little egret (*Egretta garzetta*), and the gray heron (*Ardea cinerea*). The little egret and gray heron abundances were summed because of their similar requirements (i.e., they form a small functional group).

### 2.3 Statistical analyses

#### 2.3.1 Year‐to‐year analyses

We used for year‐to‐year analyses the synchrony index *η* defined by Gross et al. (2014), which is constructed as the mean cross‐correlation between each species abundance and the summed abundances of the rest of the community (Equation 1):

(1) $$\eta =\frac{1}{n}\sum_{i}\text{Corr}\left(X_{i},\sum_{j\neq i}X_{j}\right)$$

where *X_i_* is the abundance or biomass of species *i* in a community of *n* species (or more generally *n* system components) and the correlation is computed over the years. This synchrony index varies between −1 (perfect compensation, total abundance is constant) and 1 (complete synchrony), while 0 represents a case where populations fluctuate independently on average. Contrary to other indices (e.g., Loreau and de Mazancourt (2008)’s *ϕ*), this index is independent from the richness *n* of the community (Blüthgen et al., 2016; Hallett et al., 2016). This is particularly important here as we perform analyses at different taxonomic scales, and therefore with a different *n* in Equation 1. All analyses performed with abundance in the main text are performed with biomass in Appendix S4.

We computed the synchrony index *η* over all available years, but separately for cold and warm seasons, using the codyn package in R (Hallett et al., 2016). That is, we constructed two community‐level time series of species abundances, one for the cold season and one for the warm season. To do so, we averaged monthly bird abundances, for each species, over the season duration. In follow‐up analyses, we also differentiated periods before and after 2006, given that a management change occurred within the reserve in 2006. We considered both the synchrony within a given guild (e.g., among species of the *Calidris* genus) or between guilds (e.g., between the summed abundances of the 7 species of tribe *Anatini* and the sum of the 6 *Calidris* species). In the latter case of between‐guild comparisons, we summed species together before seasonal averaging, to consider seasonal averages of the monthly guild‐level abundance. Finally, we computed *η* within the community of the 60 most frequent birds.

We computed the statistical significance of the synchrony index by comparing the observed values to the distribution of *η* under the null hypothesis (Gouhier & Guichard, 2014), which amounts to cross‐correlations of value zero between species abundances (or guild‐level abundances, when considering taxonomic or functional groups). The challenge, to construct such null hypothesis, is to remove all cross‐correlations while keeping the exact same autocorrelation in each individual time series. Therefore, for each set of time series, we constructed 1000 surrogates in which we kept auto‐correlations but removed cross‐correlations between time series. There are multiple ways to erase cross‐correlations depending on the resolution of the considered community. Within guilds, we shifted the time series (Purves & Law, 2002) while between guilds (two groups only), we used a frequency‐based approach (Iterative Amplitude‐Adjusted Fourier Transform or IAAFT, see Schreiber & Schmitz, 2000). We first explain the shift‐based approach: The suite of abundance values (after seasonal averaging) is displaced by a random temporal lag *τ*, so that a value *y_t_* is now found at *y_t+τ_*. At the boundary (the end of the time series), remaining points are displaced toward the beginning of the time series, which implements a toroidal shift. This method works well when comparing many time series corresponding to the multiple species. However, when computing synchrony across only two groups (between guilds), spurious cross‐correlations could emerge with a shift‐based approach as the number of possible combinations is more limited. Therefore, to test for synchrony between the summed abundances of two guilds or taxonomic units, we used the more sophisticated IAAFT method (Schreiber & Schmitz, 2000), which retains the frequency spectrum of the time series while randomizing its values. We obtained 1000 sets of randomized time series for each computed synchrony index. We then compared the number of *η*_H0_ values which exceeded or were inferior to the observed value to compute the p‐value (North et al., 2002): we use the ratio (*r* + 1)/(*n* + 1) where *r* is the number of surrogate values that are ≥*η*_obs_ or ≤*η*_obs_, and *n* is the number of surrogates. Independence of species was rejected at the 10% significance threshold with a Benjamini–Hochberg correction, as we compare across 2 seasons and 3 periods (all years, before 2006, after 2006), with partially overlapping data. This was found satisfactory based on simulated data, although power is low for detecting compensation (i.e., the null cannot always be rejected) when only two groups are compared (Appendix S5).

#### 2.3.2 Wavelet analyses

In addition to the time‐domain analyses above, we performed wavelet analyses at multiple temporal scales, ranging from a month to several years. Wavelet analyses provide information on community synchrony for a given temporal scale or frequency, and a given location in time along the time series. This was performed at the whole community level, including the 60 most frequent bird species, and for the rich wader and waterfowl communities, as well as the group formed by the great cormorant, gray heron and little egret. All wavelet analyses take as input the monthly time series data. Based on the work by Keitt (2008) and follow‐up by Vasseur et al. (2014), we used the wavelet modulus ratio to measure the synchrony between time series.

(2) $$\rho \left(t,s\right)=\frac{\int_{‐\infty }^{+\infty }\frac{1}{\sqrt{2\pi }}e^{‐\frac{1}{2}{\left(\frac{\tau ‐t}{s}\right)}^{2}}\left|\sum_{i}w_{i}\left(\tau ,s\right)\right|d\tau }{\int_{‐\infty }^{+\infty }\frac{1}{\sqrt{2\pi }}e^{‐\frac{1}{2}{\left(\frac{\tau ‐t}{s}\right)}^{2}}\sum_{i}\left|w_{i}\left(\tau ,s\right)\right|d\tau }$$

where *w_i_* (*t*, *s*) is the continuous Morlet wavelet transform of species *i* at time *t* for scale *s*, and |·| is the modulus of the complex number. The numerator considers the total abundance variation $\left|\sum_{i}w_{i}\left(\tau ,s\right)\right|$ at a given temporal scale *s* and location in time *τ*, while the denominator considers a weighted sum of the fluctuation amplitude of each species $\left(\sum_{i}\left|w_{i}\left(\tau ,s\right)\right|\right)$. The Gaussian weights in the numerator and denominator ensure that *ρ* (*t*, *s*) is specific to time *t* and scale *s*. This index *ρ* is close to 0 when species (or compartments) compensate and reaches 1 when they are synchronous (Keitt, 2008). Significance of high and low values of *ρ* was evaluated using a 10% overall level. The null hypothesis was constructed using the IAAFT (Schreiber & Schmitz, 2000), using 1000 surrogate time series, and computing the corresponding *ρ* values for each one (similar to Cazelles et al., 2014). The robustness of the wavelet approach to the presence of exactly zero values is tested in Appendix S6. Appendix S7 and S8 further test the ability of *ρ* to identify compensation or synchrony in cases of skewed species abundance distribution, either in the mean or in the amplitude of temporal variation.

Statistical significance testing was always done using a significance level *α* = 10%, which was based on our previous experience working with (statistically short) ecological time series and analyses of numerical simulations using *α* = 10%, provided in Appendix S5‐S8.

All computer codes for statistical analyses, and the datasets, are available in a GitHub repository https://github.com/fbarraquand/BirdTimeSeries_Teich and stored at Zenodo (Picoche et al., 2022). Finally, we want to highlight a conceptual issue worth keeping in mind: both *ρ* and *η* indicate synchrony when reaching one, but such synchrony should be understood as the reciprocal of compensation rather than exactly synchronized peaks and troughs for all species (i.e., phase synchrony). Unlike phase synchrony, compensation and community‐level synchrony depend on the distribution of abundance variation within the community. In the limit case where a single species abundance fluctuates more than all others combined, compensation may not even be reachable, as variation in the abundance of that dominant species cannot be offset by changes in numbers of other species. Only when species densities have commensurate temporal variability will the concepts of community‐level synchrony and phase synchrony exactly match.

## 3 RESULTS

### 3.1 Synchrony within phylogenetic or functional groups

**FIGURE 2:**
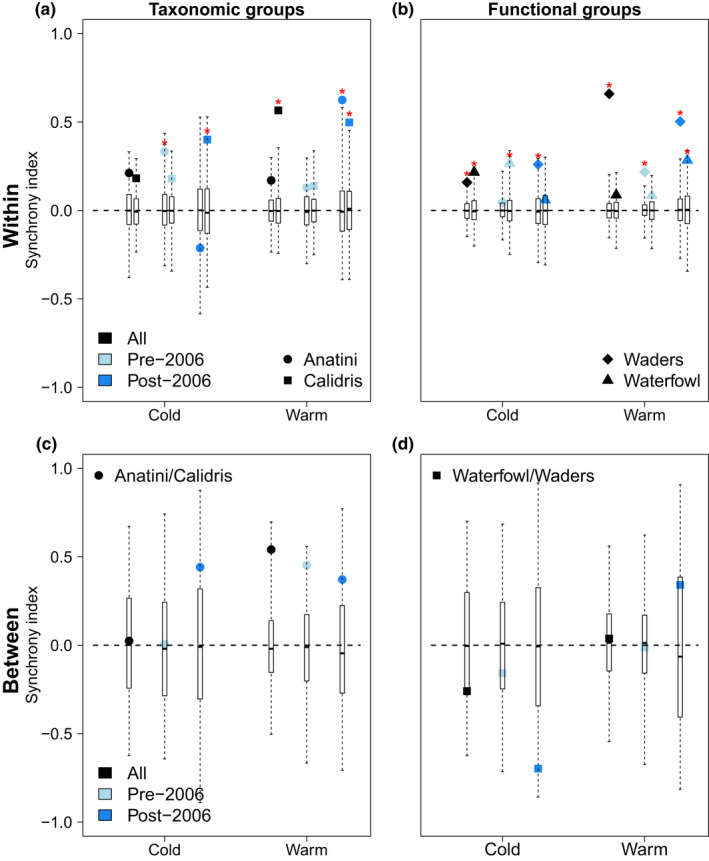
Synchrony index (*η*) as a function of the season (cold and warm seasons), calculated *within* (top, a–b) and *between* (bottom, c–d) groups. The groups considered were different taxonomic groups (*Anatini*, *Calidris*, left a–c) or functional groups (waders vs waterfowl, right b–d). The index was computed in each panel on the whole dataset (black) or using two periods: before and after 2006 (light and dark blue), the year of the change in water level management. Boxplots indicate the distribution of *η* under the null hypothesis (independent species) and filled symbols correspond to the observed values. Red stars correspond to synchrony values significantly different from the null model, at the 10% threshold with a Benjamini‐Hochberg correction

**FIGURE 3:**
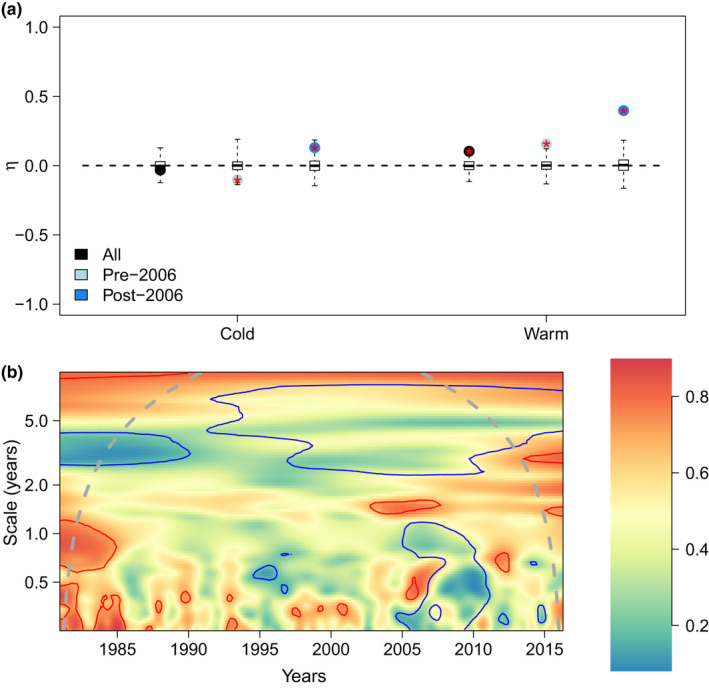
Synchrony indices for the whole community of frequently observed birds. Panel (a) presents yearly synchrony (*η*) for both seasons and (b) the wavelet modulus ratio (*ρ*). The latter index scales from 0 (compensation, blue color) to 1 (synchrony, red color). Blue and red lines respectively delineate regions of significantly lower and higher synchrony than the null model (independently fluctuating species, but conserving their original Fourier spectrum), at the 10% level

Using a taxonomic classification of the community, focusing on the genera *Calidris* and tribe *Anatini* (formerly *Anas*) as two key examples of taxonomic units with contrasted preferences, within‐genus synchrony dominates year‐to‐year analyses for the two seasons (Figure 2). Using functional groups (waders and waterfowl), synchrony within functional groups was also prominent. The index *η* is indeed mostly positive, and always positive whenever it is significantly different from zero (null hypothesis of no temporal correlation between species). Therefore, there is no compensation within guilds (Figure 2a and b) across years, for the two seasons. This matches the patterns obtained within the entire wetland bird community (Figure 3a).

For the cold season, abundances within *Calidris* and *Anatini* display opposite changes in synchrony values in response to the management change in 2006, with species within *Anatini* becoming less synchronous overtime, although we should mention that these changes are not statistically significant. For the warm season, the management change, which consisted of lowering the water levels, created little change in communities of species within the *Anatini* and *Calidris*: They are all synchronous.

**FIGURE 4:**
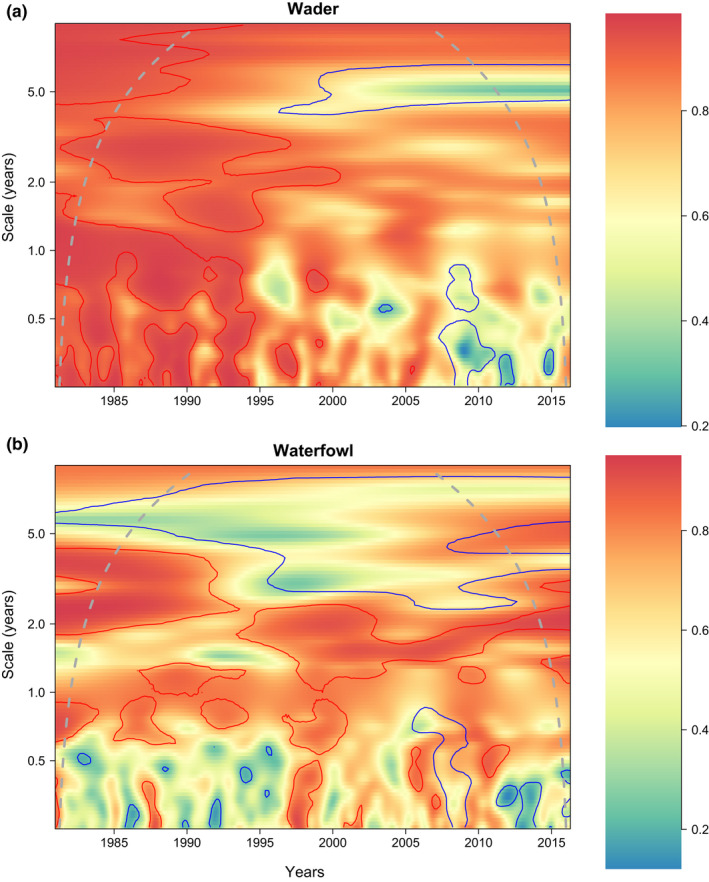
Wavelet modulus ratio (*ρ*) for (a) the wader community and (b) the waterfowl community. The index *ρ* scales from 0 (compensation, blue color) to 1 (synchrony, red color). Blue and red lines respectively delineate regions of significantly lower and higher synchrony than the null model (independently fluctuating species, but conserving their original Fourier spectrum), at the 10% level

Even though there is no widespread community‐wide or genus‐wide compensation across years (separating the two seasons), there could be compensation at finer temporal scales, for example, a month or two, or coarser scales, over several years. Such compensation could also occur at specific time intervals instead of throughout the whole time series, a time‐dependency that wavelet analyses allow to reveal. When we consider the wavelet modulus ratio (Figure 4), that is, a time‐varying and scale‐dependent strength of synchrony, we can see that there is synchrony even at a fine temporal scale throughout most of the time series. However, post‐2006, there seems to be a possibility for episodic compensation on a temporal scale of approximately 2–4 months, for both waders and waterfowl. There could also be within‐guild compensation at scales of 5 years, approximately post‐2000 for waders and pre‐2005 for waterfowl. Waterfowl synchrony trends likely influence whole community trends (Figure 3).

We thus find contrasted results regarding the effect of the management change on synchrony within guilds or within the whole bird community, depending on the type of analyses. Year‐to‐year analyses yield unclear results for both guilds. At shorter (one or two months) and longer (five years) timescales though, wavelet analyses show that the management change may decrease synchrony and even promote compensation.

### 3.2 Synchrony between phylogenetic or functional groups

More easily interpretable results can be found when we examine synchrony vs compensation between functional groups (Figure 2d). As we consider only two functional or phylogenetic groups, *η* reduces to a simple correlation between two groups. *Anatini* and *Calidris* are positively correlated in the warm season (for all periods), and have unclear correlations during the cold season (Figure 2c). In contrast, waders and waterfowl are negatively correlated during the cold season and positively correlated during the warm season (Figure 2d). Although the negative correlation is not statistically significant, it is consistent for both pre‐ and post‐2006 periods.

### 3.3 Synchrony in a small module with known competition

**FIGURE 5:**
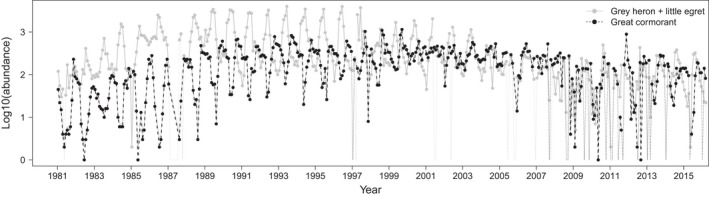
Time series of great cormorant abundance (dash‐dotted black line), as well as summed abundances of gray heron and little egret (solid gray line)

**FIGURE 6:**
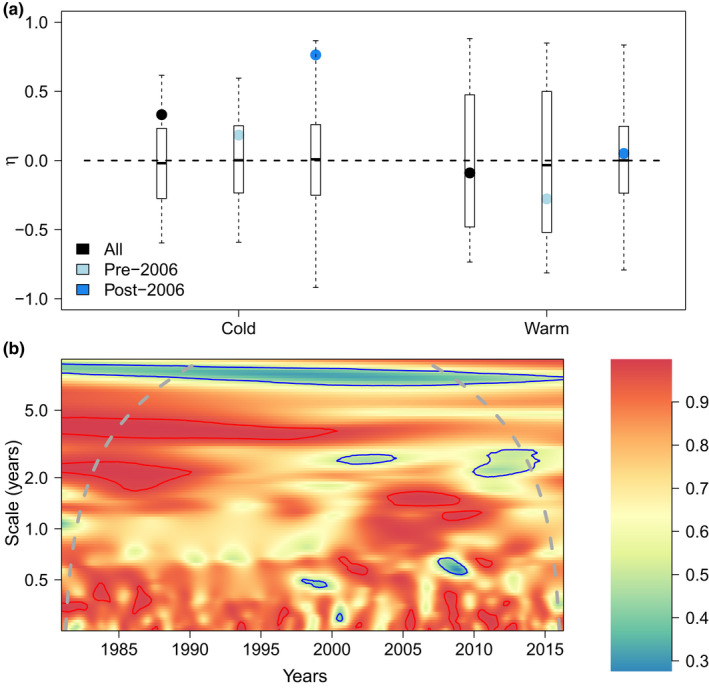
Synchrony analyses of cormorant vs egret and heron. Panel (a) presents yearly synchrony (*η*) for both seasons and (b) the wavelet modulus ratio (*ρ*). The latter index scales from 0 (compensation, blue color) to 1 (synchrony, red color). Blue and red lines respectively delineate regions of significantly lower and higher synchrony than the null model (independently fluctuating species, but conserving their original Fourier spectrum), at the 10% level

Compensation could be expected upon visual inspection of the time series of the two groups formed by cormorant on the one hand, and little egret plus gray heron (summed as a small functional group) on the other hand (Figure 5, though see Appendix S3 for alternative representations). However, we see on Figure 6 that synchrony is in fact the rule around the annual scale and below, when considering the wavelet modulus ratio. We wondered if the patterns in Figure 5 were caused by the use of a log scale, but we found that in fact the correlation was higher rather than lower on the log scale (Appendix S3). However, over long temporal scales (~8 years), we observe consistent compensation, which could correspond to the slow change in composition observed within this small community module, which was already visible on the abundance time series plot (Figure 5). There is some statistically significant compensation over shorter timescales as well, but only at very specific times. The absence of marked compensation at short temporal scales may be an inevitable consequence of the difference in the amplitude of temporal variation between the two groups (Appendix S8), as opposite annual phases for the two time series can be observed before 2000 (Figure 5).

## 4 DISCUSSION

Between‐species compensation was not found across years (for two separate seasons), synchrony between species being the rule. This was true at the whole‐community level, and *within* genera or functional groups, in year‐to‐year analyses of cold and warm seasons. Yet, summing the abundances of species within a functional group and comparing these total abundances of contrasted functional groups, it was possible to find compensation across years, during the cold season corresponding to wintering birds (although the null hypothesis of no correlation could not be rejected); that is, there was compensation *between* functional groups. Moreover, compensation did eventually appear in community and within‐guild level wavelet analyses considering longer temporal scales. These results are robust to using biomass in place of abundance (Appendix S4). A focus on a module of three species with known competition also revealed clear compensation at scales ≈8 years. We elaborate below on these findings.

### 4.1 Synchrony *within* or *between* guilds

Given that we compare the level of synchrony/compensation within guilds (with many species) and between guilds (with only a handful of groups), we checked in Appendix S5, using the dynamical model of Gross et al. (2014), if changing the number of “compartments” (*n*) in the index *η* could affect its value. It did not have marked effects, unless the number of compartments is equal to 2, in which case significance is hard to achieve and some compensatory dynamics can be missed with weak environmental response. Additionally, we found—still using this dynamical model—that if two guilds respond in opposite ways to a shared environmental driver, the stronger the response of growth rates to the driver, the lesser the compensation indicated by *η* at the whole community level. An intuitive explanation of this modeling result is that when there are two groups and many species within a group, a stronger forcing homogeneizes the dynamics within a group as much as it creates differences between groups. This might explain the low levels of compensation that we found in our empirical dataset, at the overall wetland bird community level (Figure 3), in spite of the clear presence of two guilds (waders and waterfowl) reacting in opposite way to a shared driver (here, water levels). Analyses at several taxonomic/functional scales are therefore warranted to be conclusive about compensation, which mirrors what was suggested by earlier plant studies (e.g., Bai et al., 2004). Future case studies with more than two main functional groups may be instructive, to challenge the generality of our findings.

We used correlation between the summed abundances of closely related species (species within the *Anatini* tribe vs species within the *Calidris* genus) or the summed abundances of functionally similar species (waders vs waterfowl) to uncover compensation. The functional group classification produced some compensation between guilds while the taxonomic classification did not, despite the contrasted habitat preferences of these two phylogenetic groups. Using functional groups therefore produced more logical results, although as we stressed above, the null hypothesis of no compensation in year‐to‐year data could not be rejected, which may be due to the low power of the test when comparing two groups.

We expected to see compensation at the functional group scale for both cold and warm seasons. The separation of seasons allowed to differentiate summer residents (some of whom may be breeding) and wintering birds, to remove the overwhelming influence of the seasonal migratory cycle. In both of those seasons, we had reason to expect waders and waterfowl to have different environmental preferences. Instead, waders and waterfowl were found to correlate negatively only during the cold (wintering) season. A simple explanation is that the reserve might be closer to its carrying capacity for these species in winter, so that space is limited and increases in one functional group are compensated by decreases in the other. The dominant species in each guild (Figure 1), such as *C*. *alpina* for waders and *A*. *crecca* for waterfowl, are migratory species which are much more abundant in winter than summer in that area, which adds to the plausibility of the reserve reaching carrying capacity. Of course, the space constraint should not be taken too literally: birds are obviously mobile and do forage outside of the reserve (e.g., waders moving to the nearby Arcachon bay mudflats), but there are costs to those movements (energetics, mortality risk due to nearby hunting) which make the reserve a very attractive wintering site where birds both rest and forage to some degree. Packing even more birds over its 120 ha may just not be feasible, so that increases in one guild result in decreases in the other. Compensation might therefore be easier to detect during the cold season because the study area is “filled,” and it is not detected in our warm season (May to August) because there are less birds overall.

It may be better to say that we detected “compensation” rather than “compensatory dynamics” between bird species (Gonzalez & Loreau, 2009), if compensatory dynamics is thought to result from births and deaths, that is, population dynamics. Indeed, the observed long‐term changes in species composition (more waders, proportionally less waterfowl; Appendix S2) is likely due to an increased inflow of birds preferring low water levels (waders), and outflow of birds preferring high water levels (waterfowl), under an overall space constraint (at least in winter, as we explained above). Bird settlement decisions for both winter and spring/summer seasons are the proximal causes of bird species composition in the reserve, rather than local population dynamics. However, it would be incorrect to conclude that because the local compensation in winter that we found results from bird behavior, it is disconnected from regional‐scale community dynamics: which species are present in the reserve—safe from hunting—affects ultimately their survival and reproductive success, which then feeds back into the regional‐scale community dynamics.

### 4.2 Effect of the change in management on synchrony

Although we performed a first set of analyses using the whole time series, we have also performed year‐to‐year analyses pre‐ and post‐2006. The reason for these additional analyses is that a marked change in management occurred around 2006, after which the water levels were lower. Separating pre‐/post‐2006 and comparing to the previous analyses allows to disentangle the effect of the “normal” dynamics from the effect of this management change. Pre‐ and post‐2006 analyses showed very little differences with whole time series analyses for either the warm or the cold season. However, in the wavelet modulus ratio analyses, we see at monthly or 5‐year timescales more compensation after 2006 within waders; this could reflect that the community is becoming saturated with waders. The effects of disturbances on the level of synchrony or compensation are likely idiosyncratic: for instance, Keitt (2008) found increased synchrony after disturbance while van Klink et al. (2019) found no clear effect.

### 4.3 Synchrony in a small module with known competition

We now focus on the cormorant‐heron‐egret module, for which we knew beforehand that competition for resting and roosting sites in the summer season occurs between, on the one hand, great cormorants, and on the other hand, little egrets and gray herons (C. Feigné, *pers. obs.*). Abundance time series suggested some negative correlation, but it was not found in year‐to‐year analyses for which synchrony (or an absence of relation) dominates. Instead, we find that compensation mostly occurs on a scale of 8 years, much above the annual scale, which is a likely consequence of the slow shift in frequencies of cormorants and little egrets/gray herons. The reason why we do not find a compensation at the monthly to annual scale pre‐2000 in spite of some opposition of annual phases may be related to the large difference in the amplitude of short‐term temporal variation between the two groups. When one functional group or species dominates the temporal variation, as shown in Appendix S8, its dominance of temporal variation can forbid the occurrence of compensation since by definition no increase in the numbers of the species that fluctuate less may compensate for the decrease in the species that fluctuates more (and vice versa).

### 4.4 Conclusion and perspectives for theory

Overall, our results suggest to search for compensation more often *between* rather than *within* functional groups, and over relatively long timescales, above the typical temporal autocorrelation of the dominant driver (e.g., above 5 years if the main driver is a seasonal climate). This rejoins the recent findings of van Klink et al. (2019) who found that increased functional differences between species tend to decrease synchrony in beetles, and earlier results of Bai et al. (2004) on negative covariation of plant functional groups. Our suggestion goes against calls to search for compensation within closely related species but at very short timescales (Gonzalez & Loreau, 2009; Vasseur & Gaedke, 2007), below the timescale of the main synchronizing seasonal environmental driver, to filter out precisely its synchronizing effect. Searching for compensation at temporal scales below the seasonal abiotic driver (e.g., temperature) was partly motivated by studies on plankton whose population dynamics are usually much faster than the dominant abiotic driver, with short generation times, so that the effects of competition may be manifest at the scale of a few weeks or months.

In theory, we could have expected compensation to manifest also at the smallest temporal scale of our survey (monthly). Indeed, the community dynamics in our case are driven by the movements and settlement decisions of birds, reacting to perceived food and space availability, rather than by births and deaths directly. Such behavioral dynamics can certainly be much faster than bird population dynamics and could operate at the scale of weeks or months. However, such compensation due to short‐term movements was not observed except perhaps in some years. We suspect that because many species share common abiotic drivers (e.g., disturbances due to nearby hunting, local climatic conditions) fluctuating even within a single season, their dynamics can be synchronized by these drivers at monthly temporal scales. It is noteworthy that even in planktonic systems, the temporal scale of compensation has often been found to be well above that of the forcing driver (Brown et al., 2016; Keitt, 2008). Thus, our findings reinforce previous suggestions to search for compensation over relatively long timescales (several years for vertebrates or plants).

The attractor of community dynamics, that is the shape of community trajectories in phase space, seems to be more or less an annual cycle here: The dominant species fluctuate seasonally, but even though there are shifts in some species dynamics, no abundant species seem to exhibit violent multi‐year oscillations. If we had to describe our community mathematically, a dynamical model with a stable fixed point forced by seasonality and some noise would probably be appropriate. This mild fluctuation scenario somehow contrasts with the dynamics of other communities, such as insect pests, that have quite often multi‐year cycles (on top of seasonal cycles, for multivoltine species), with possibly strong indirect interactions between similar species mediated by predators and parasitoids (Murdoch et al., 2003). In the latter context of internally generated variability (“Endogenous compensatory cycles” in Gonzalez & Loreau, 2009), compensation may be more likely: Klapwijk et al. (2018) recently reported only transient synchrony between species of moths, that typically exhibit such multi‐year fluctuations.

In many ways, searching for abundance compensation using biodiversity time series data is searching for needles in a haystack: Only some specific temporal and functional/taxonomic scales allow to see compensation whilst numerous confounding factors make the community co‐vary positively at all other scales (Vasseur et al., 2014). When a common species fluctuates much more than the rest, this can also lessen or forbid compensation. Thus, although the knowledge of specific biological mechanisms increasing the densities of some species at the expense of others can help, synchrony will likely dominate community‐level time series data for closely related species, even in species that compete strongly (Loreau & de Mazancourt, 2008; Ranta et al., 2008). This is true even in cases of known mechanisms of competition for space or shifts in community composition due to abiotic changes affecting differentially species preferences, as in this study. We therefore suggest that “zooming out” functionally (considering summed abundances of dissimilar functional groups) and temporally (considering temporal scales well above the periodicity of the dominant abiotic driver) may often be the best strategy to see the compensation that will inevitably manifest at some scales, if the community‐level biomass is to be maintained within bounds in the long run.

## CONFLICT OF INTEREST

The authors have no conflicts of interests to declare.

## AUTHOR CONTRIBUTIONS

**Frédéric Barraquand:** Conceptualization (equal); Data curation (equal); Formal analysis (equal); Investigation (equal); Methodology (lead); Project administration (lead); Supervision (lead); Validation (equal); Visualization (supporting); Writing — original draft (lead); Writing—review & editing (lead). **Coralie Picoche:** Data curation (supporting); Formal analysis (equal); Investigation (equal); Methodology (supporting); Software (lead); Validation (equal); Visualization (equal); Writing—original draft (supporting); Writing—review & editing (supporting). **Christelle Aluome:** Data curation (equal); Investigation (supporting); Software (equal); Validation (equal); Visualization (equal); Writing—review & editing (supporting). **Laure Carassou:** Conceptualization (equal); Project administration (equal); Supervision (equal); Validation (supporting); Visualization (supporting); Writing—original draft (supporting); Writing—review & editing (supporting). **Claude Feigné:** Conceptualization (equal); Data curation (supporting); Funding acquisition (lead); Investigation (lead); Resources (equal).

## Supporting information

Appendix S1‐S8

## Data Availability

All the code and data used for analyses are available at https://github.com/fbarraquand/BirdTimeSeries_Teich and archived at Zenodo, https://doi.org/10.5281/zenodo.6368332 (Picoche et al., 2022) and the main data file deposited at Dryad https://doi.org/10.5061/dryad.zpc866t9v.
