# Supplementary material for: Looking for compensation at multiple scales in a wetland bird community
Source: Ecol Evol. 2022 Jun 2;12(6):e8876. doi: 10.1002/ece3.8876 (PMC9163198; doi:10.1002/ece3.8876)
Supplement: Supplementary file 1 — Appendix S1‐S8 [file ECE3-12-e8876-s001.pdf]

Online Supporting Information

Appendices to *Looking for compensation at multiple scales in a wetland bird community.* Barraquand F., Picoche C., Aluome C., Carassou L., and Feigné C., Ecology and Evolution. DOI: 10.1002/ece3.8876.

Appendix S1 - List of species

Among the 279 bird species which were observed throughout the monitoring, we focused on 2 functional groups (waterfowl and waders) and 2 phylogenetic groups (*Anatini, Calidris*) described in Table 1. In Table 2, we also present the other most frequent birds in the reserve, in addition to the two main functional groups. The rest of the community (not detailed here) amounts to 1% of the total number of observed birds.

Functional group	Species	Name	% occurrence	% abundance
Waterfowl	Anas acuta	Northern pintail	59.9	1.708
	Anas crecca	Eurasian teal	64.2	6.614
	Anas platyrhynchos	Mallard	63.8	3.388
	Mareca penelope	Eurasian wigeon	36.5	0.055
	Mareca strepera	Gadwall	48.7	0.308
	Spatula clypeata	Northern shoveler	63.5	1.580
	Spatula querquedula	Garganey	26.0	0.032
	Anser anser	Greylag goose	47.3	1.135
	Aythya ferina	Common pochard	57.1	1.053
	Aythya fuligula	Tufted duck	45.8	0.467
	Aythya marila	Greater scaup	6.5	0.005
	Branta bernicla	Brant	2.3	0.001
	Branta canadensis	Canada goose	5.1	0.002
	Cygnus olor	Mute swan	50.0	0.618
	Fulica atra	Eurasian coot	64.3	16.007
	Netta rufina	Red-crested pochard	27.0	0.030
	Tadorna tadorna	Common shelduck	53.1	0.870
Waders	Actitis hypoleucos	Common sandpiper	25.3	0.054
	Arenaria interpres	Ruddy turnstone	14.7	0.020
	Calidris alba	Sanderling	4.1	0.004
	Calidris alpina	Dunlin	37.2	26.394
	Calidris canutus	Red knot	22.6	0.406
	Calidris ferruginea	Curlew sandpiper	14.8	0.067
	Calidris minuta	Little stint	25.1	0.129
	Calidris pugnax	Ruff	11.4	0.010
	Calidris temminckii	Temminck's stint	4.6	0.001
	Charadrius dubius	Little ringed plover	18.2	0.040
	Charadrius hiaticula	Common ringed plover	34.2	0.921
	Gallinago gallinago	Common snipe	23.1	0.184
	Himantopus himantopus	Black-winged stilt	12.1	0.079
	Limosa lapponica	Bar-tailed godwit	18.4	0.327
	Limosa limosa	Black-tailed godwit	29.7	1.802
	Numenius arquata	Eurasian curlew	17.0	1.167
	Numenius phaeopus	Whimbrel	11.1	0.102
	Pluvialis apricaria	European golden plover	5.0	0.002
	Pluvialis squatarola	Grey plover	32.9	0.669
	Recurvirostra avosetta	Pied avocet	25.0	0.775
	Tringa erythropus	Spotted redshank	23.9	0.133
	Tringa glareola	Wood sandpiper	9.6	0.008
	Tringa nebularia	Common greenshank	25.0	0.173
	Tringa ochropus	Green sandpiper	15.4	0.014
	Tringa totanus	Common redshank	32.4	0.670
	Vanellus vanellus	Northern lapwing	22.8	0.265

Table 1: Composition of the two main functional groups considered in the manuscript. The genera on which we focused for our taxonomy-based comparisons are written in colour. The percentage of occurrence corresponds to the number of dates when a species was observed, compared to the total number of observation dates. The percentage of abundance corresponds to the ratio of the abundance of a given species to the total abundance of all birds observed during the monitoring.

Functional group	Species	Name	% occurrence	% abundance
Other	Accipiter nisus	Eurasian sparrowhawk	12.8	0.003
	Alcedo atthis	Common kingfisher	12.1	0.017
	Ardea alba	Great egret	30.0	0.150
	Ardea cinerea	Grey heron	63.1	2.480
	Bubulcus ibis	Western cattle egret	37.9	0.381
	Chroicocephalus ridibundus	Black-headed gull	51.3	9.645
	Ciconia ciconia	White stork	51.3	0.905
	Circus aeruginosus	Western marsh harrier	23.3	0.017
	Egretta garzetta	Little egret	55.1	6.511
	Falco peregrinus	Peregrine falcon	13.8	0.003
	Gallinula chloropus	Common moorhen	42.2	1.106
	Larus argentatus	European herring gull	36.4	3.414
	Larus fuscus	Lesser black-backed gull	32.1	0.088
	Larus marinus	Great black-backed gull	38.9	0.039
	Larus michahellis	Yellow-legged gull	17.5	0.210
	Milvus migrans	Black kite	12.8	0.106
	Nycticorax nycticorax	Black-crowned night heron	58.4	0.160
	Phalacrocorax carbo	Great cormorant	57.7	3.616
	Platalea leucorodia	Eurasian spoonbill	48.2	0.720
	Podiceps cristatus	Great crested grebe	28.6	0.033
	Podiceps nigricollis	Black-necked grebe	12.0	0.008
	Rallus aquaticus	Water rail	40.4	0.594
	Tachybaptus ruficollis	Little grebe	44.8	0.403
	Threskiornis aethiopicus	African sacred ibis	12.5	0.019

Table 2: Most frequent birds (observed more than 75 times during the monitoring) which are not described in the previous functional groups (see previous legend for the meaning of the column headers).

Appendix S2 - Temporal patterns of the Teich reserve bird community

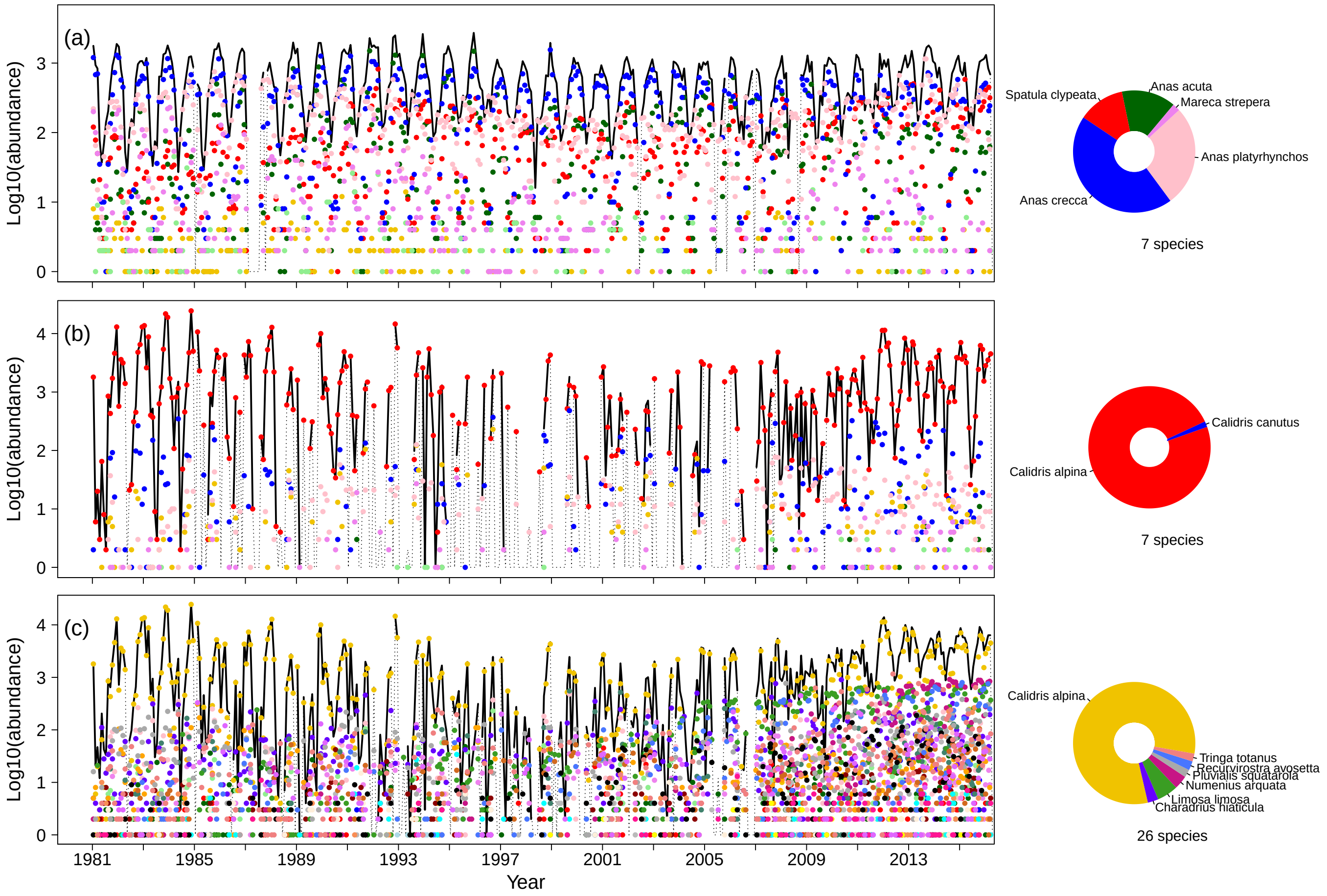

Figure S1: Time series of counts for ducks of the tribe *Anatini* (a), calidrids (b, *Calidris* genus), and all waders (c, including calidrids). The solid black lines represent how summed abundances change over time for each guild when abundances are strictly positive, while thin dotted lines connect positive to zero abundances. The coloured symbols below the curves represent each species abundances. Species composition on the donut plots (right) are given for species whose relative abundance is over 1% in the group considered. The total number of species constituting the group is given below the donut plot.

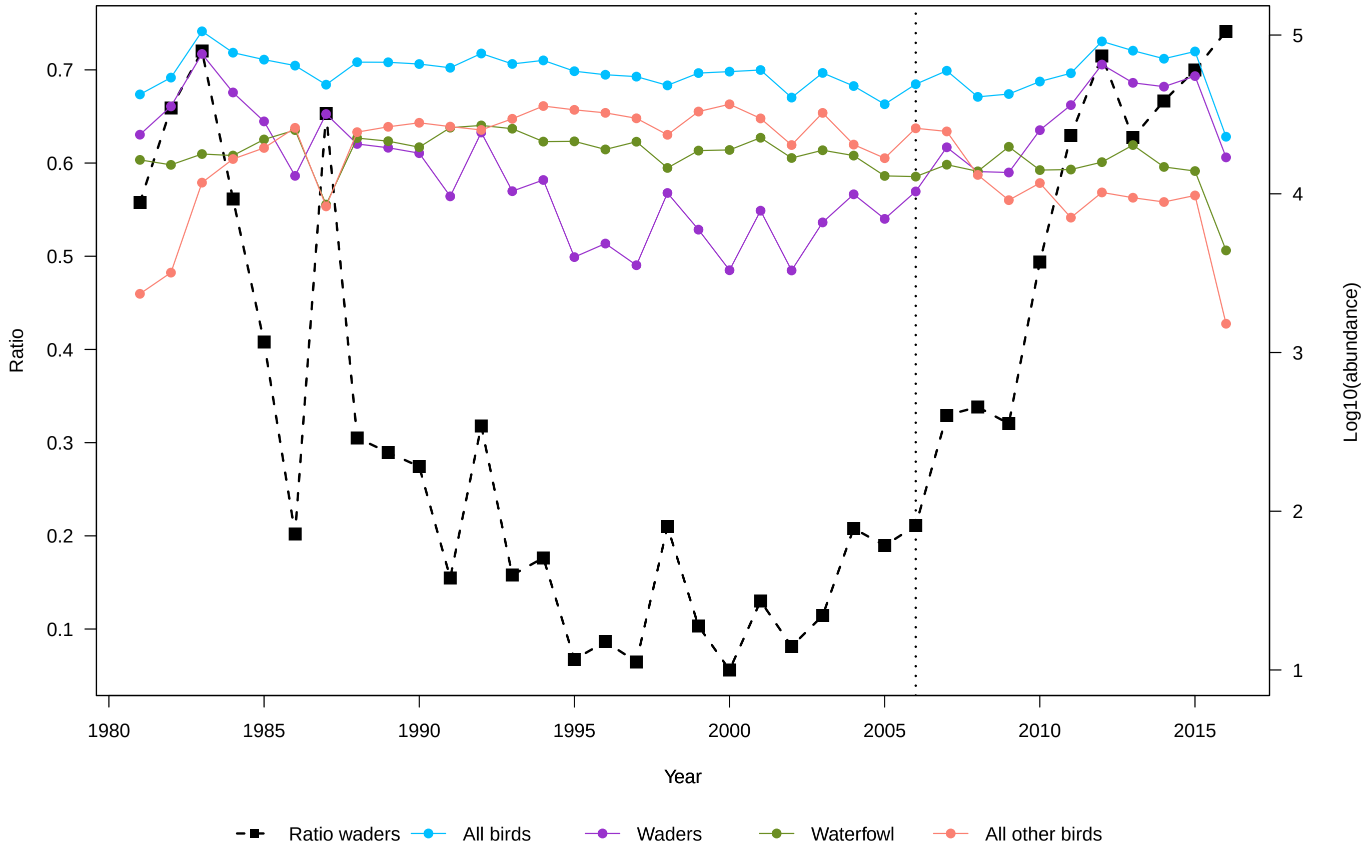

Figure S2: Temporal trends in the abundance of all birds and in the main functional groups (waders and waterfowl), with one point per year. The waterfowl category actually includes all species functionally similar to ducks (ducks, geese and swans, i.e. anatids, and the common coot). The term "All other birds" corresponds to the sum of all individuals neither belonging to waders nor to waterfowl. The dashed line presents the ratio of wader individuals in the whole community, i.e., their relative abundance, which changes markedly over time.

Appendix S3 - Additional plots concerning cormorant and heron + egret

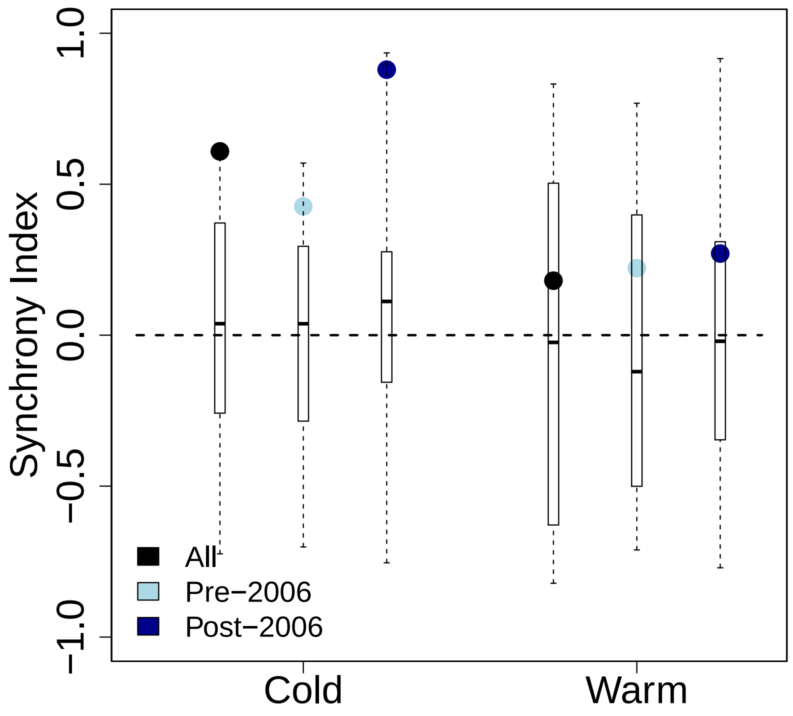

Figure S3: Gross' synchrony index as a function of the season (cold and warm seasons), calculated on log-transformed abundance data for the two groups formed by cormorant and grey heron + little egret. The index was computed in each panel on the whole dataset (black) or using two periods: before and after 2006 (light and dark blue), the year of the change in water level management.

We noted on Fig. 5 in the main text that cormorants, herons and egrets seemed to compensate each other, at least for the first period of the time series. This compensation was seen on a log scale, and seemed conspicuous on that scale. We thus wondered if log-transforming the abundance would affect the values of the synchrony index observed for this group, and make compensation more visible. It appears to be the reverse: synchrony values are higher with log-transformed abundances (none of them being significant at a 10% threshold). In fact, an annual representation of abundances (seasonal averages, Fig. S4) makes the correlation between the two groups less obvious, which is probably why it only appears at larger scales (>5 years).

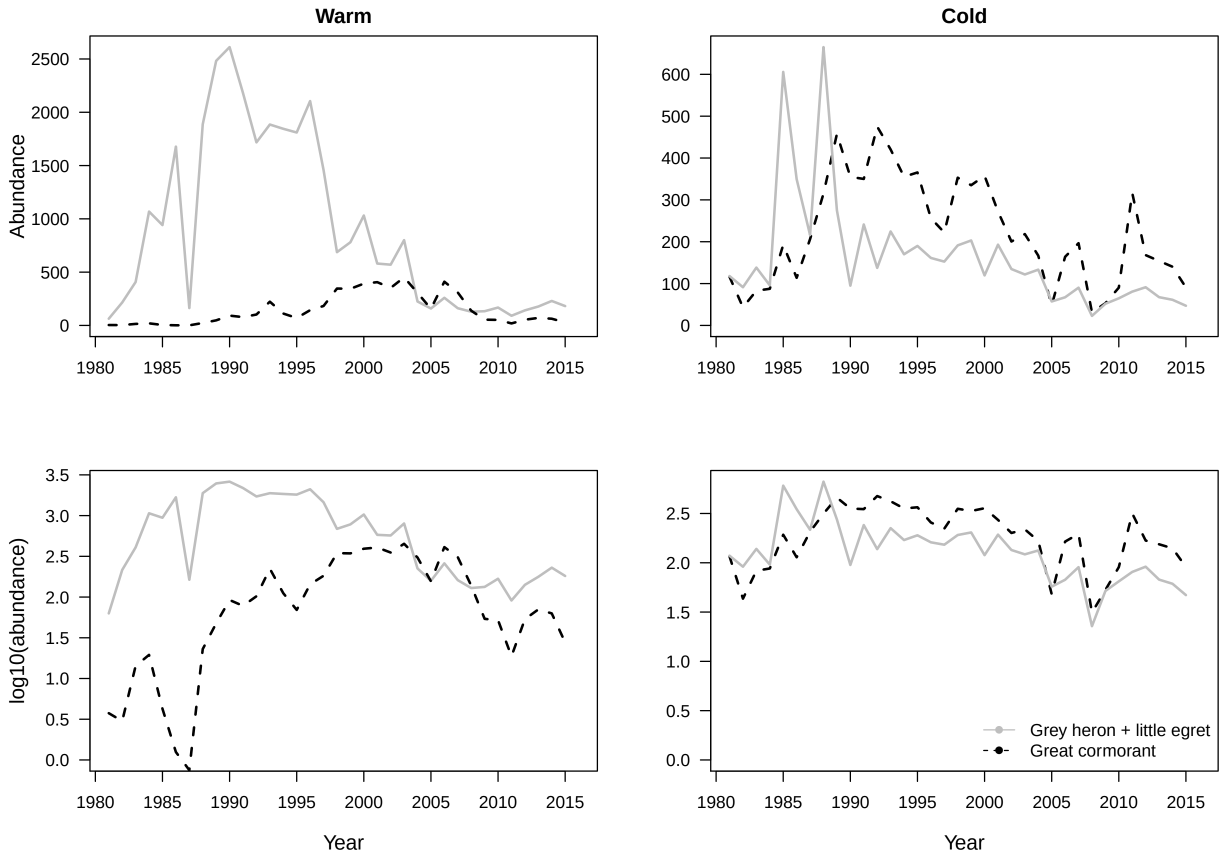

Figure S4: Time series of seasonally averaged abundances of the great cormorant (dashed black line) and the grey heron plus little egret group (solid grey line), differentiating between warm and cold seasons.

Appendix S4 - Synchrony indices using biomasses

We used the mean body mass of each species to compute observed biomasses from abundances and eventually the corresponding synchrony indices. This change in the state variable did not change our conclusions.

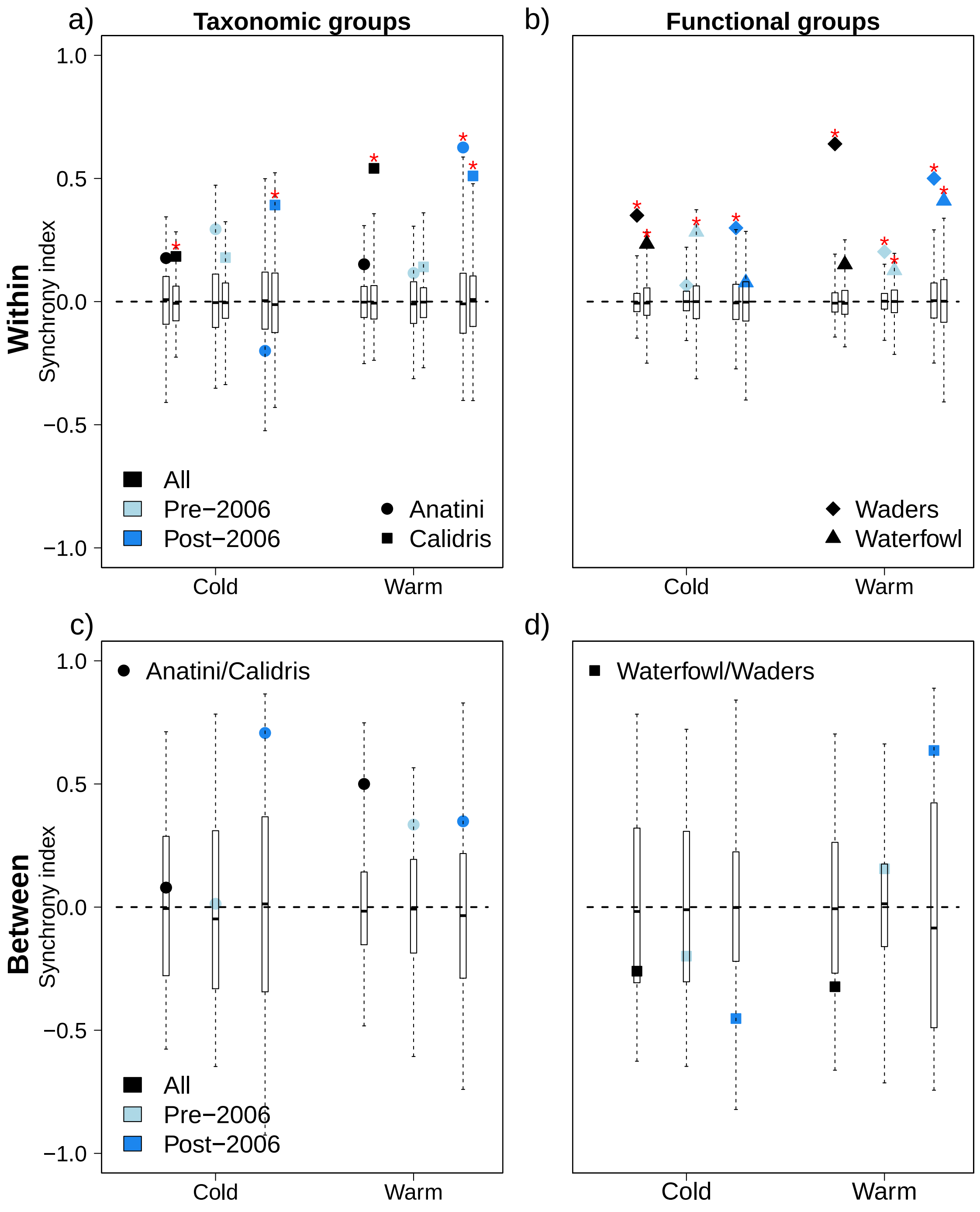

Figure S5: Gross' synchrony index (η) as a function of the season (cold and warm seasons), calculated within (top, a-b) and between (bottom, c-d) groups, using biomasses instead of abundances. The groups considered were different functional groups (waterfowl - i.e. ducks, geese and swans - vs waders, right b-d) or taxonomic groups (*Anatini* tribe, *Calidris* genus, left a-c) groups. The index was computed in each panel on the whole dataset (black) or using two periods: before and after 2006 (light and dark blue), the year of the change in water level management. Boxplots indicate the distribution of η under the null hypothesis (independent species) and filled symbols correspond to the observed values. Red stars correspond to synchrony values significantly different from the null model, at the 10% threshold with a Benjamini-Hochberg correction.

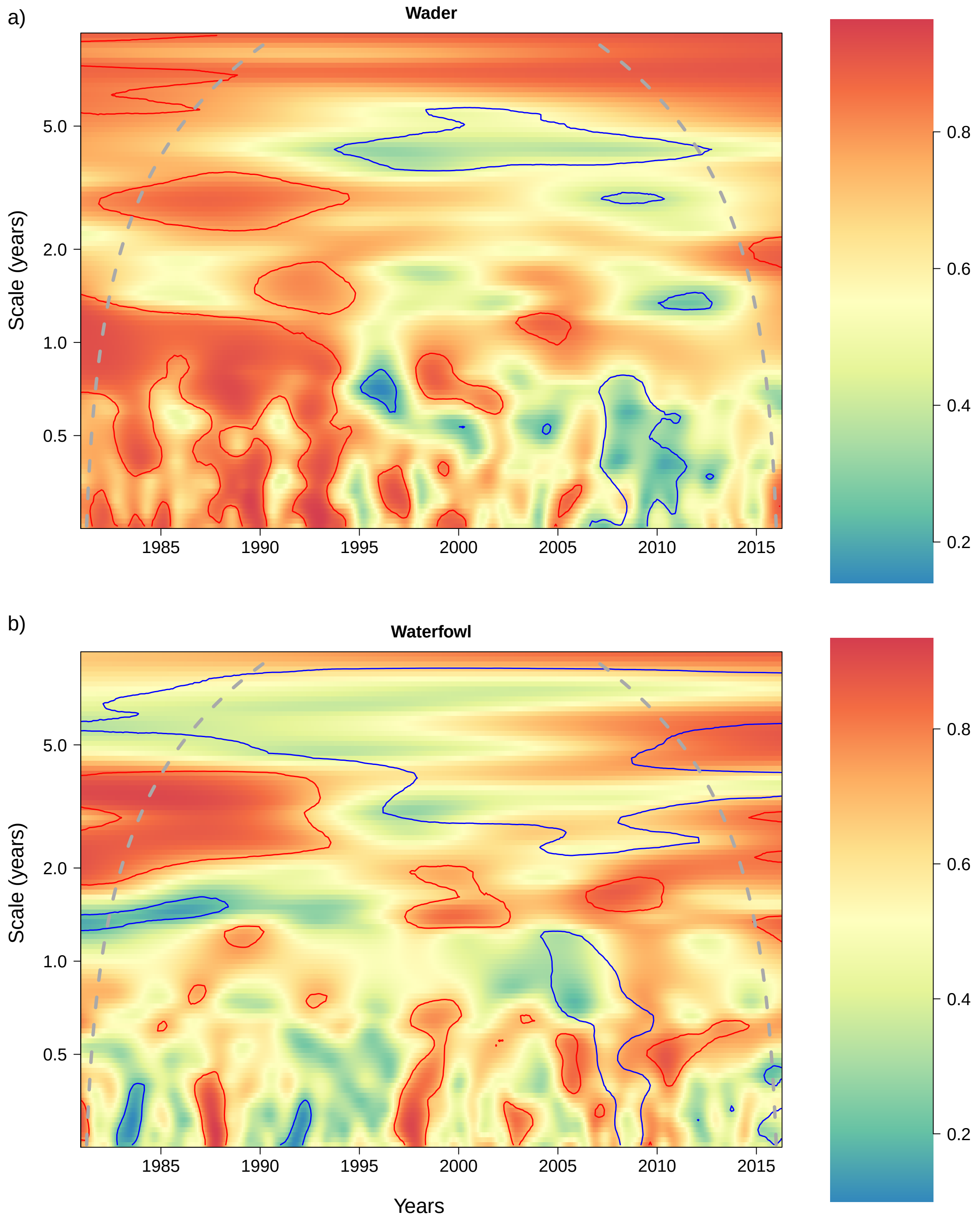

Figure S6: Wavelet modulus ratio for the wader and waterfowl community, scaling from 0 (compensation, blue color) to 1 (synchrony, red color), using the mean biomasses instead of the abundances of each species. Blue and red lines respectively delineate regions of significantly lower and higher synchrony than the null model (independently fluctuating species with the same Fourier spectrum), at the 10% level.

Appendix S5 - Properties of the Gross *et al.* (2014) whole community synchrony index η, when two groups react in opposite ways

Here, we make η vary with the number of species, richness of the community, and the strength of the effect of the environment. Starting from the model developed by Gross *et al.* (2014) in their Appendix D, we explored the effect of a shared environmental driver on a community formed by two groups reacting in opposite ways to this driver.

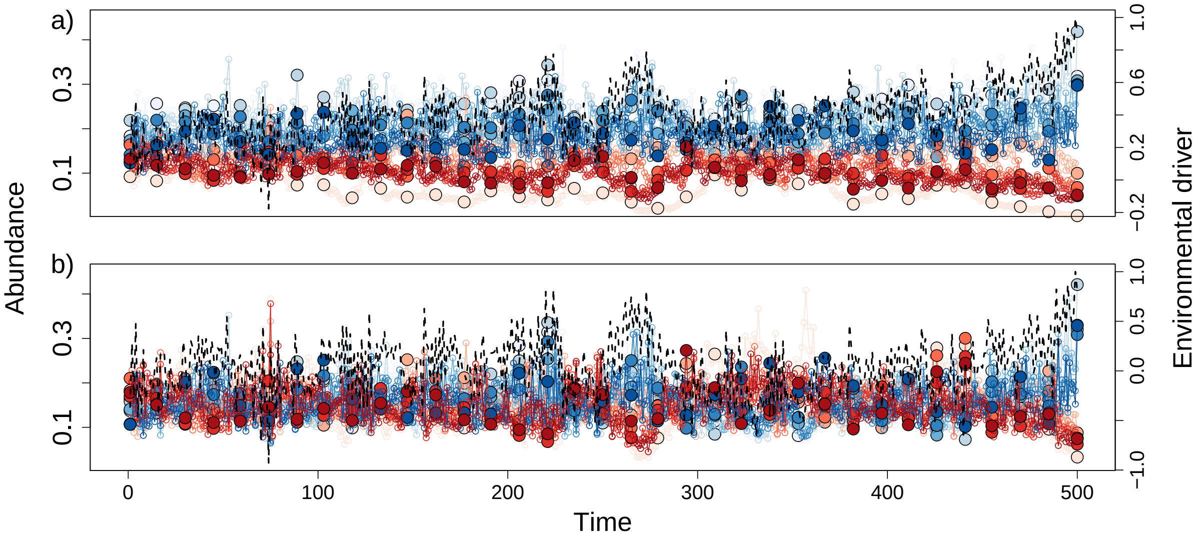

Figure S7: Time series for a community of 10 species, with a strong effect of the environment (b=0.75, see eq. 1) and a time series of 500 time steps. Open circles represent all points of the time series while large filled circles correspond to the sub-sampling of the time series (keeping only 35 points). Shades of blue indicate species which react positively to the environmental signal and shades of red indicate species which have a negative reaction to the same signal. The environmental signal is shown in black, dashed lines, and indexed on the right axis. This signal either follows an increasing trend (a), or is just autocorrelated (b).

We assume that the environmental driver $U(t)$ is an autocorrelated signal (see below for details).

The dynamics of species i then follows the equation 1:

$$x_i(t+1) = x_i(t)\exp\left(r_i\left(1 - \frac{x_i(t) + \sum_{j\neq i}^{n} \alpha x_j(t)}{K}\right) + b_i U(t) + \epsilon_i(t)\right) \quad (1)$$

where the whole community is formed by $N = 2n$ species, with 2 groups of n species, each group having a reaction to the environmental driver opposite to that of the other group, that is $\forall i \in [1,n], \exists j \in [1,n], b_j = -b_i = b$. The growth rate r_i follows a normal distribution with mean 1 and standard deviation 0.25. All interaction coefficients α are set to 0.5 and $K = \frac{1+\alpha(N-1)}{N}$, to keep the model in other ways exactly similar to Gross *et al.* (2014). The noise $\epsilon_i(t)$ is normally distributed, centered on 0 with a standard deviation of 0.1.

We compared results for time series of length 35 (the length of our data set when computing η), 100 and 500. For all simulation experiments, the dynamics are first run for 500 time steps as a burn-in. To take into account different observational designs, we either take the first 35 or 100 time steps of the following 500-time steps series, or subsample the time series in order to get 35 or 100 time points (which removes some autocorrelation in the dynamics). We also considered several community richness (2, 10, 30 and 60, to remain close to the number of species in the different groups we analysed), and several strengths of the response to the environmental signal ($b =$[0.1, 0.5, 0.75]). For each combination of parameters, we computed 10 repetitions (i.e., replicates).

We considered different types of environmental driver (simply autocorrelated or with a linear trend), crossed with subsampling / no subsampling as described above. We considered in total three scenarios, and present below how an increasing strength of response to the environment changes expectations for all three:

- Scenario 1: $U_t = u_t$ where u_t is an autocorrelated signal (standardized); no subsampling of the data

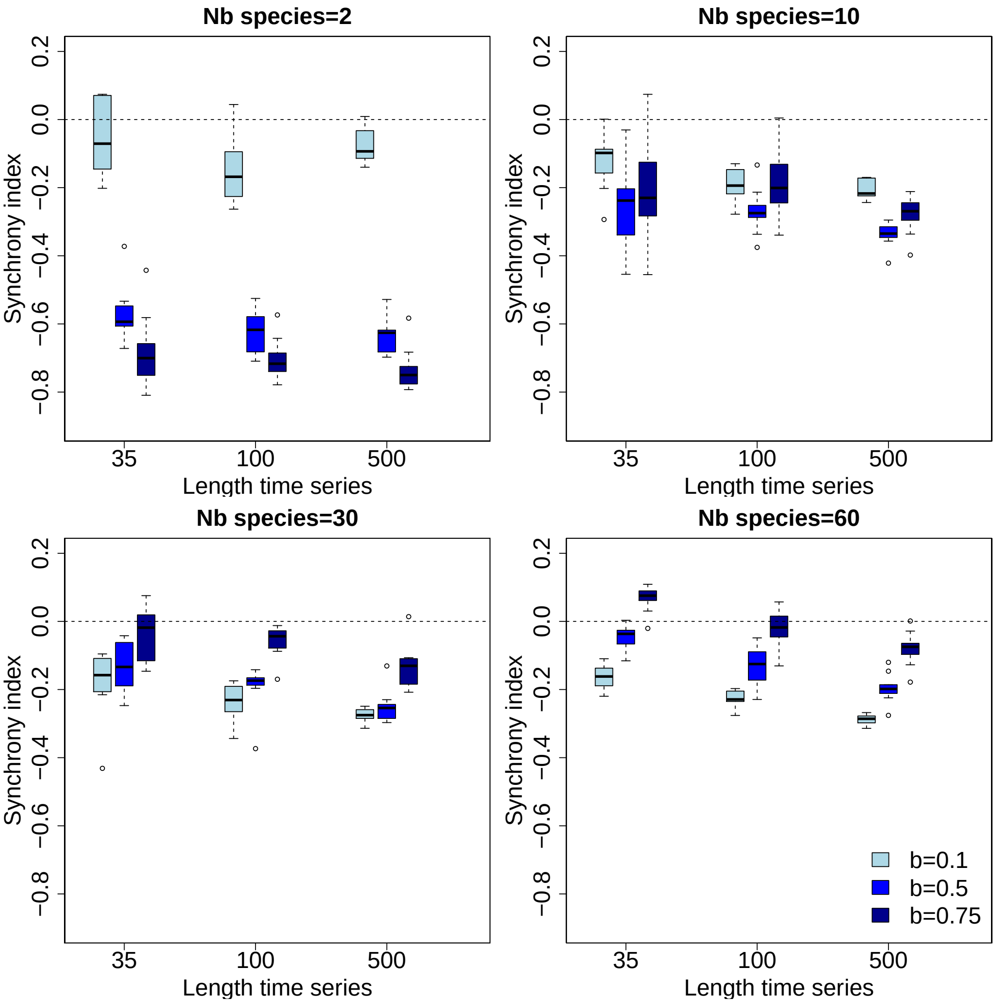

Figure S8: Gross *et al.* (2014)'s synchrony index for different time series lengths and numbers of species in the community, in simulations where there is no trend in the environmental signal and the data is not subsampled, keeping the autocorrelation of the environment. The parameter b is the strength of the response to the environmental signal.

- Scenario 2: $U_t = u_t$; data subsampling

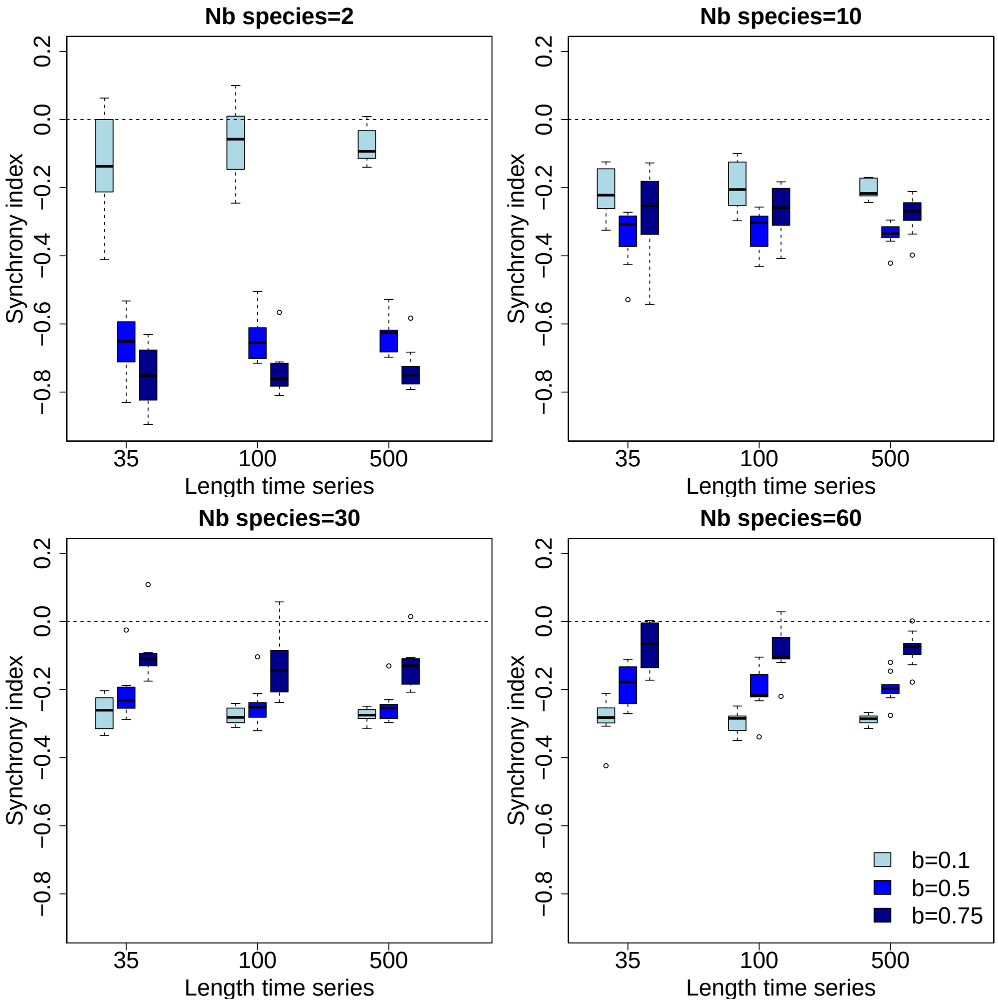

Figure S9: Gross *et al.* (2014)'s synchrony index for different time series lengths and numbers of species in the community, in simulations where there is no trend in the environmental signal and the data is subsampled (keeping 35 or 100 time steps), removing in effect the autocorrelation of the environment. The parameter b is the strength of the response to the environmental signal.

- Scenario 3: $U_t = U_{\min} + (U_{\max} - U_{\min})(u_t + x_t)/2$ where $x_t \in [0, 1]$ follows an increasing trend; data subsampling

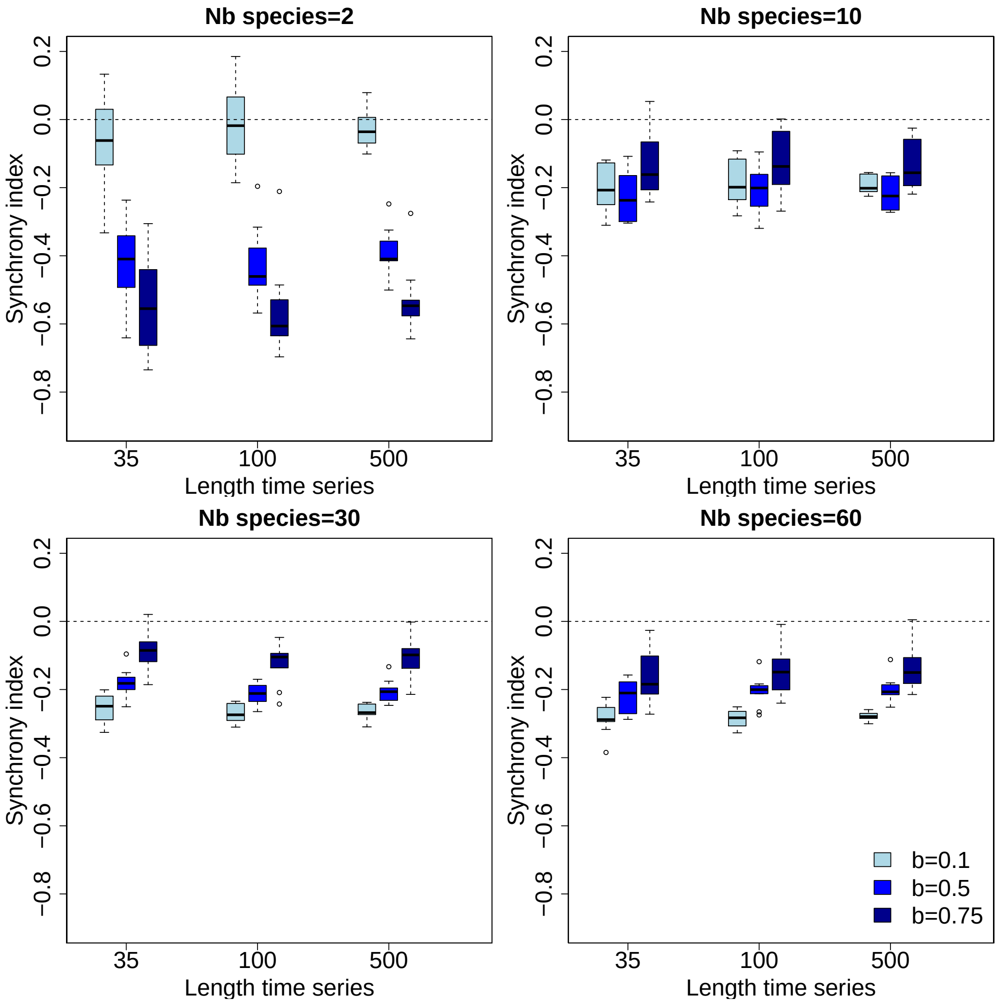

Figure S10: Gross *et al.* (2014)'s synchrony index for different time series lengths and numbers of species in the community, in simulations where there is an increasing trend in the environmental signal and the data is subsampled (for 35 or 100 time steps), removing part of the autocorrelation of the environment in the dynamics of the species. The parameter b is the strength of the response to the environmental signal.

We first confirm that unless there is a very high temporal autocorrelation in the driver (as in Fig. S8), if we consider two groups which have opposite reactions to the same driver, the Gross *et al.* (2014) index is not sensitive to the length of the time series or to the number of species in the community, in the sense that it will not indicate false synchrony. The only contradiction to this observation appears when the driver has only a small effect on the community, and the Gross *et al.* (2014) index is actually a simple correlation (i.e., the community is made of 2 species). The main finding of these analyses, aside from the robustness of the η index, is that for large communities (over 10 species), synchrony is always higher when the response to the driver is stronger. Coming back to the interpretation of our results in the Teich reserve case, this means

that the more bird population growth rates respond to changes in the water levels, the less we can expect compensation at the whole community level, even though compensation may be present between functional groups (here, groups constituted of species responding similarly to the environmental variable within each group). That said, compensation between groups in the case of a small effect of the environment can also be difficult to detect if there are only two functional groups.

Appendix S6 - Effect of zero values on wavelet analyses

We investigate here if exactly-zero abundances can distort the compensation patterns evidenced by the wavelet coherence. We chose a simulation with 10 species and 10% zero values for all species (close to a worst-case scenario). The results show that while the statistical significance of the values can change (i.e., the data is more compatible with the null hypothesis of time series uncorrelated between species, assuming the null is true), the occurrence of compensation (blue values in Fig. S11) does not. The wavelet coherence index is therefore robust to the presence of zeroes in the time series.

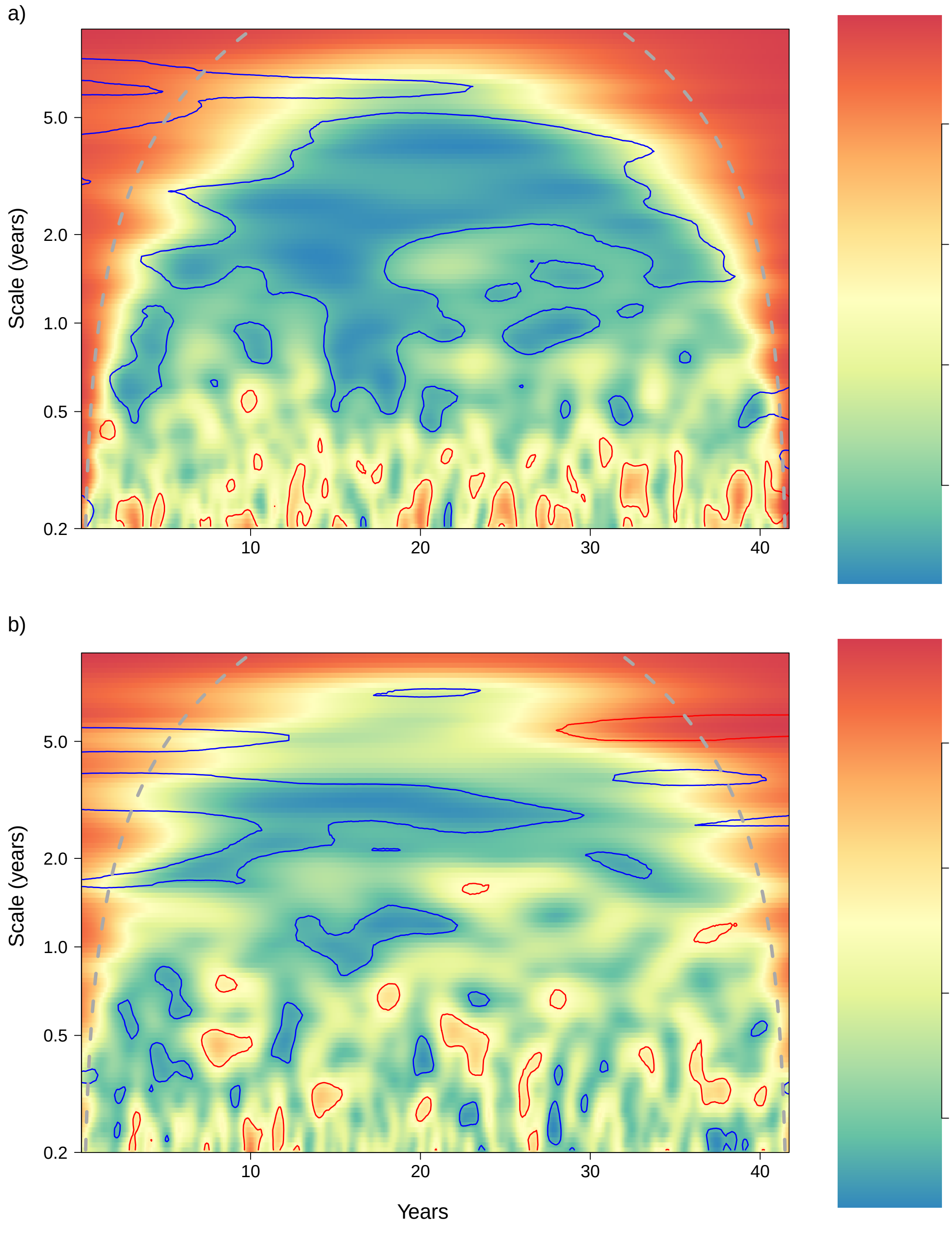

Figure S11: Wavelet modulus ratio for a 10-species community with compensatory dynamics without (a) and with (b) exactly zero values. Blue and red lines respectively delineate regions of significantly lower and higher synchrony than the null model (independently fluctuating species, but conserving their original Fourier spectrum), at the 10% level.

Appendix S7 - Effect of species dominance on wavelet analyses

Skewed species abundance distributions (SAD) might make compensation difficult to detect, as the wavelet modulus ratio ρ may be affected mostly by the abundance of the dominant species. In order to test the sensitivity of this metric to species dominance, we modeled small (4 species) and large (40 species) communities with a skewed SAD and a similar seasonal forcing, mimicking observed data. We then performed analyses with and without normalisation (i.e., standardisation).

The simulations were based on $\mathbf{x}_t \sim \mathcal{N}(\boldsymbol{\mu}_t, \boldsymbol{\Sigma})$ where the average abundance μ_i of a species is drawn from a lognormal distribution (kept constant over time) to obtain a skewed SAD, and the abundance $\mu_{t,i}$ at time t follows $\mu_{t,i} = \mu_{\min,i} + (\mu_{\max,i} - \mu_{\min,i}) \left(1 + 0.5 \sin\left(\frac{2\pi t}{P}\right)\right)$ with $\mu_{\min,i} = 0.1\mu_i$ and $\mu_{\max,i} = 0.9\mu_i$ and $P = 12$ (we need monthly abundances with a seasonal trend). The variance-covariance matrix $\Sigma_{ij} = \rho_{ij}\sigma_i\sigma_j$ with $\sigma_i = c\mu_i$ and $\rho_{ij} = 2B_{ij} - 1$ with $B_{ij} \sim \text{Beta}(a, b)$, with different (a, b) per scenario, allowing either compensation or synchrony. In this way, we can control the correlation structure while keeping a realistic mean-variance scaling for abundances. Time series for compensation or synchrony are presented for the 4-species communities in Fig. S12.

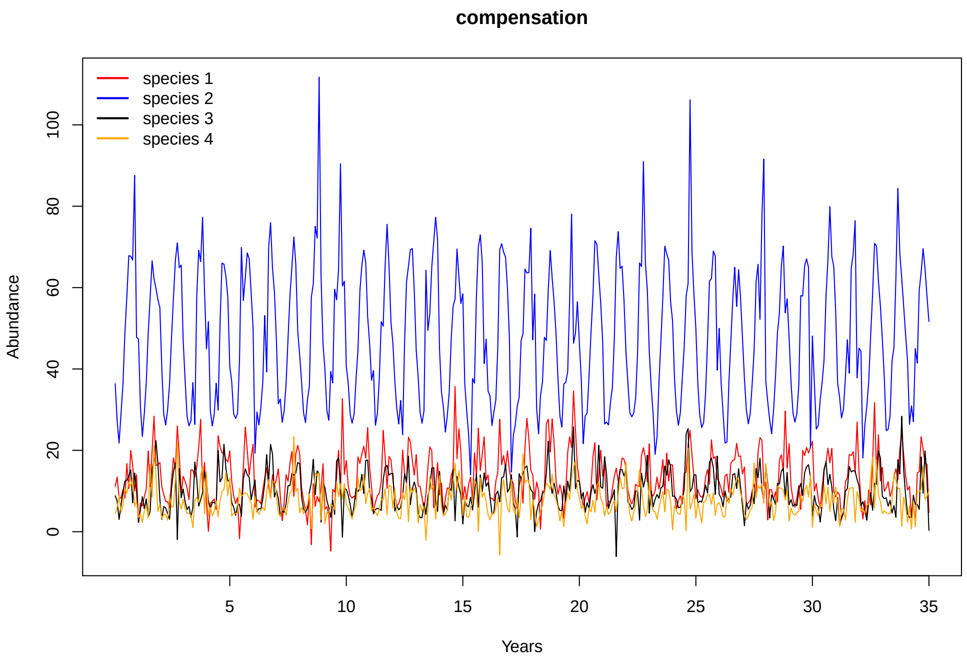

(a) Compensation

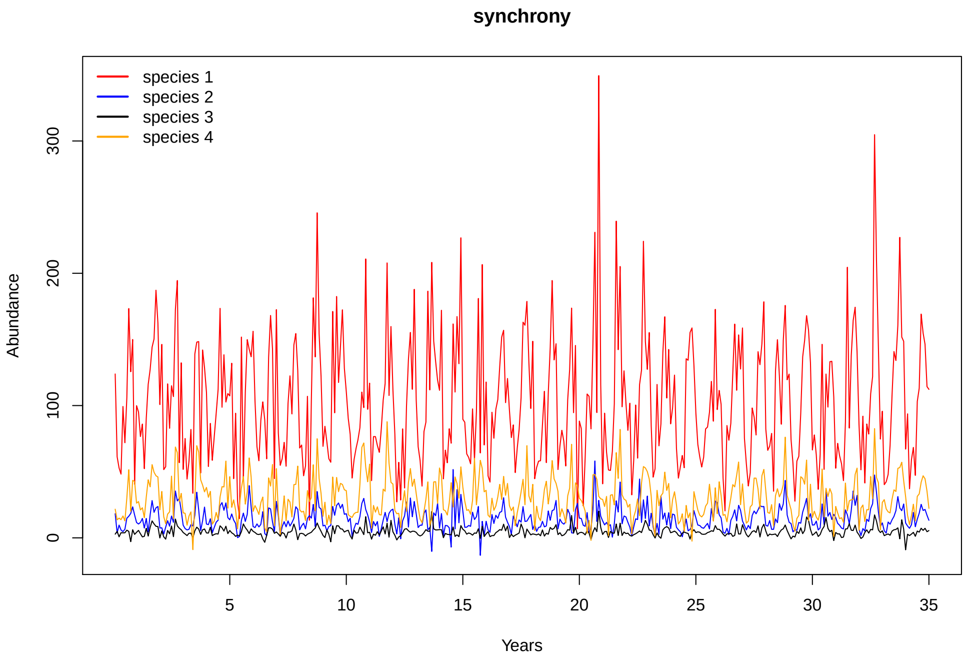

(b) Synchrony

Figure S12: Time series of communities with a skewed SAD, for 4 species, with (a) compensation ($B_{ij} \sim \text{Beta}(2, 4)$) and (b) synchrony ($B_{ij} \sim \text{Beta}(4, 2)$).

Captions for the 3 following figures:

Figure S13: Gross et al. index η and wavelet modulus ratio ρ, for 4 species with compensation, with (1) untransformed and (2) scaled time series. In a), the boxplots show the distribution of η values under the null hypothesis and red stars correspond to synchrony values significantly different from the null model, at the 10% threshold. In b), blue and red lines respectively delineate regions of significantly lower and higher synchrony than the null model (independently fluctuating species, but conserving their original Fourier spectrum), at the 10% level.

Figure S14: Gross et al. index η and wavelet modulus ratio ρ, for 4 species with synchrony, with (1) untransformed and (2) scaled time series. In a), the boxplots show the distribution of η values under the null hypothesis and red stars correspond to synchrony values significantly different from the null model, at the 10% threshold. In b), blue and red lines respectively delineate regions of significantly lower and higher synchrony than the null model (independently fluctuating species, but conserving their original Fourier spectrum), at the 10% level.

Figure S15: Gross et al. index η and wavelet modulus ratio ρ, for 40 species with compensation, with (1) untransformed and (2) scaled time series. In a), the boxplots show the distribution of η values under the null hypothesis and red stars correspond to synchrony values significantly different from the null model, at the 10% threshold. In b), blue and red lines respectively delineate regions of significantly lower and higher synchrony than the null model (independently fluctuating species, but conserving their original Fourier spectrum), at the 10% level.

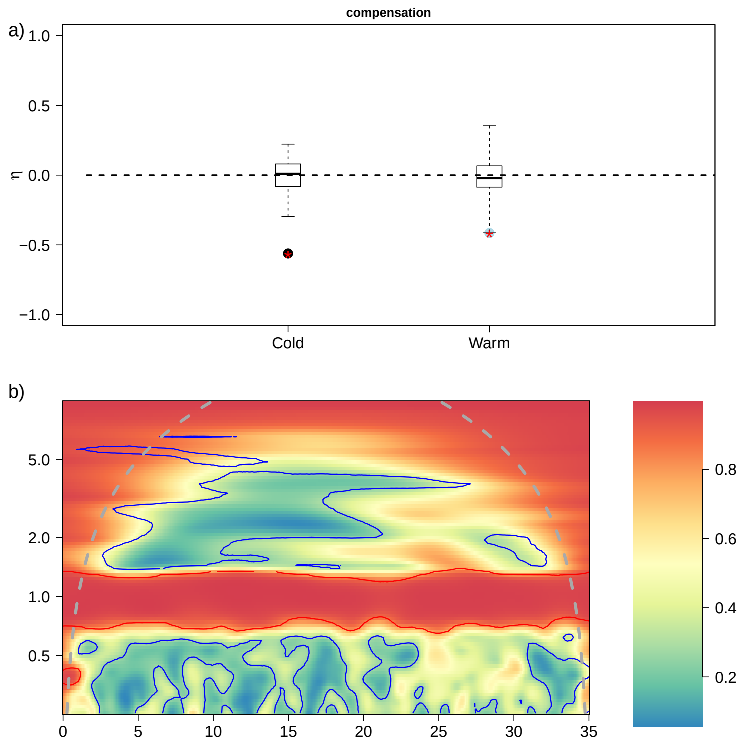

(1) Untransformed

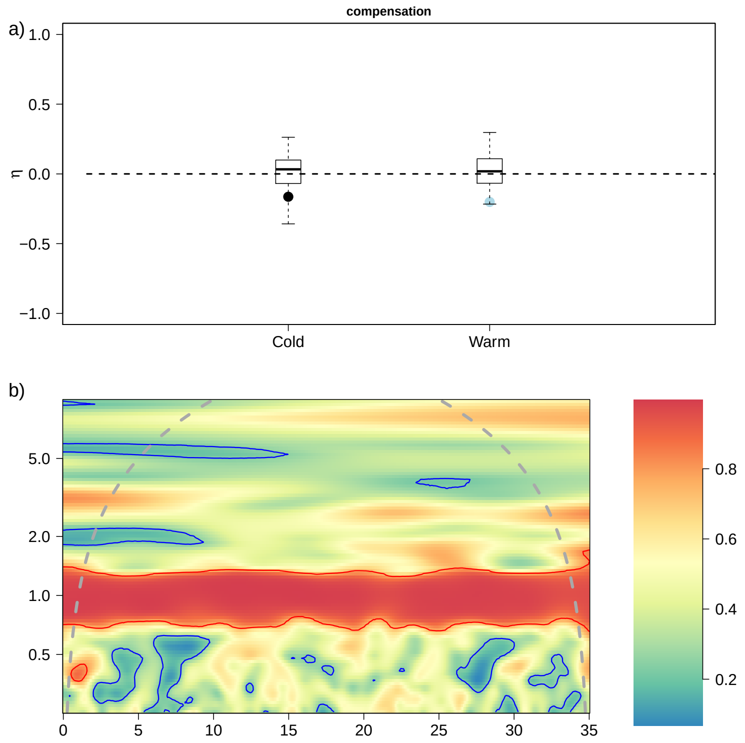

(2) Scaled

Figure S13

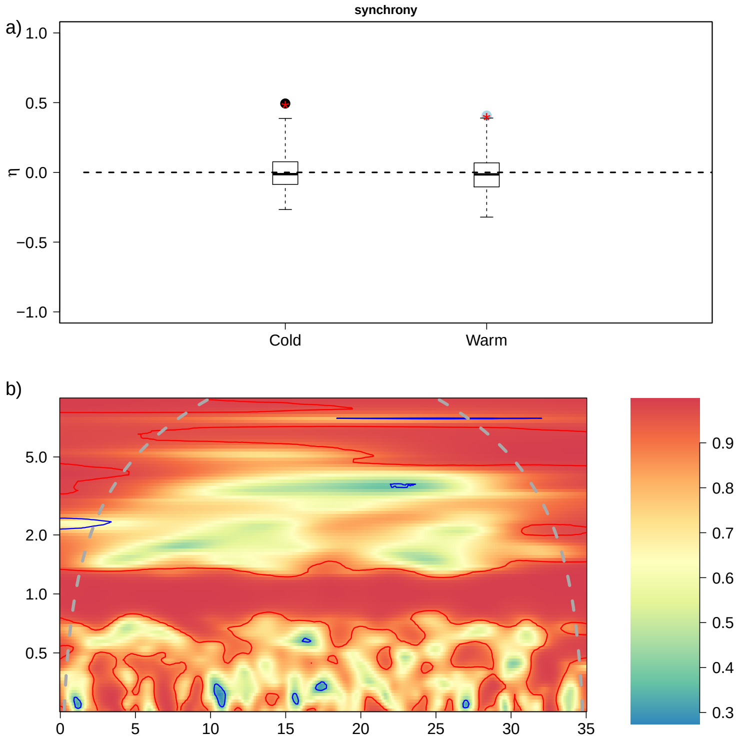

(1) Untransformed

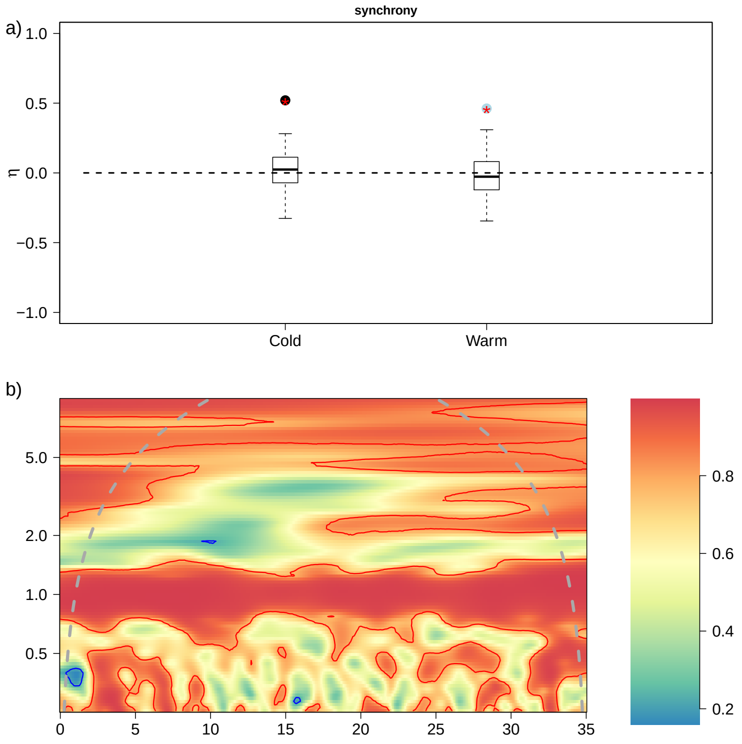

(2) Scaled

Figure S14

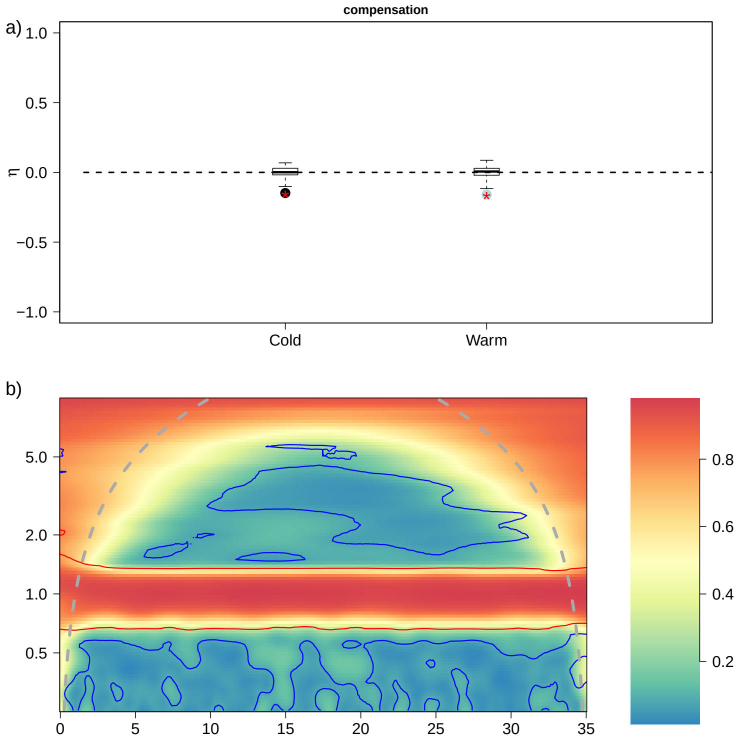

(1) Untransformed

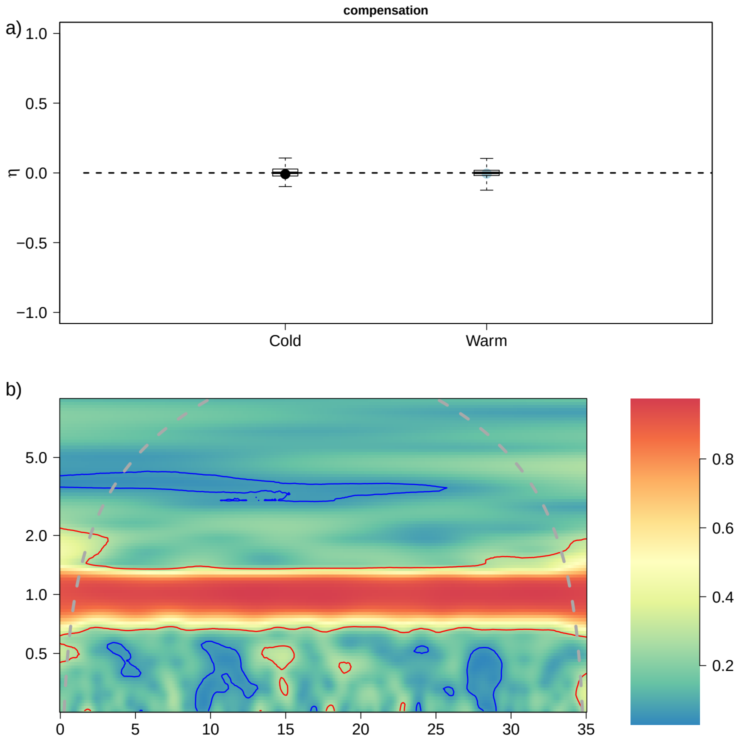

(2) Scaled

Figure S15

The fact that one species dominates does not seem to be a problem for the index to find synchrony or compensation (Figs S13, S14, S15). Normalising the time series, which was suggested by a referee, was found to decrease the statistical significance of short-term compensation (Figs. S13, S15). We stress that analyses of such scaled data essentially consider another compensation/synchrony concept than the one we modelled, more closely related to phase synchrony.

Dynamics including long-term compensation

Previous simulations included compensation only at the monthly scale, i.e. the shortest time scale in the dataset. We wondered if compensation could be detectable through wavelet-based index ρ at longer time scales, i.e., above the year. This is especially relevant in our context given that our dataset seems to have long-term trends. Also, the previous simulations showed some transfer of compensation from short to long time scales—we wondered if the reverse can be observed as well.

In a 4-species community, we forced the 2 most abundant species to have opposite long-term dynamics, with cycles of compensation over multiple years. Assuming the expected abundance of species 1, the most abundant, at time 1 is $\mu_{1,1} = \mu_a$ and the expected abundance of species 2, the second most abundant, is $\mu_{1,2} = \mu_b$, we want the expected abundances at half cycle to be $\mu_{t_{\text{cycle}/2},1} = \mu_b$ and $\mu_{t_{\text{cycle}/2},2} = \mu_a$, and to go back to their initial value at the end of the cycle, i.e. $\mu_{t_{\text{cycle}},1} = \mu_a$ and $\mu_{t_{\text{cycle}},2} = \mu_b$. In our example, we modeled 4 cycles in 35 years, i.e. the period was around 9 years. We use a model similar to the one presented in Appendix S7. Over the first half of the cyle, the dynamics follow:

$$\begin{cases} \mu_{t,1} = & \left(\mu_a + (\mu_b - \mu_a)\frac{t}{t_{\text{cycle}/2}}\right)\left(1 + 0.5\sin\left(\frac{2\pi t}{P}\right)\right) \\ \mu_{t,2} = & \left(\mu_b + (\mu_a - \mu_b)\frac{t}{t_{\text{cycle}/2}}\right)\left(1 + 0.5\sin\left(\frac{2\pi t}{P}\right)\right) \\ \mu_{t,3/4} = & \mu_{3/4}\left(1 + 0.5\sin\left(\frac{2\pi t}{P}\right)\right). \end{cases}$$

Abundances revert to their initial value at the end of the cycle. In this way, we include the synchrony due to the seasonality, but expect to detect compensation.

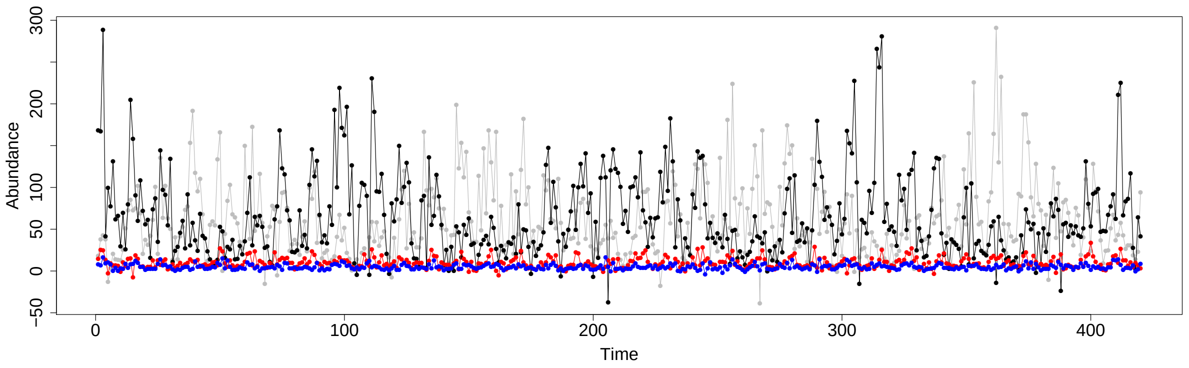

Figure S16: Time series of abundances in a 4-species community with compensation between the 2 most abundant species with a 9-year cycle.

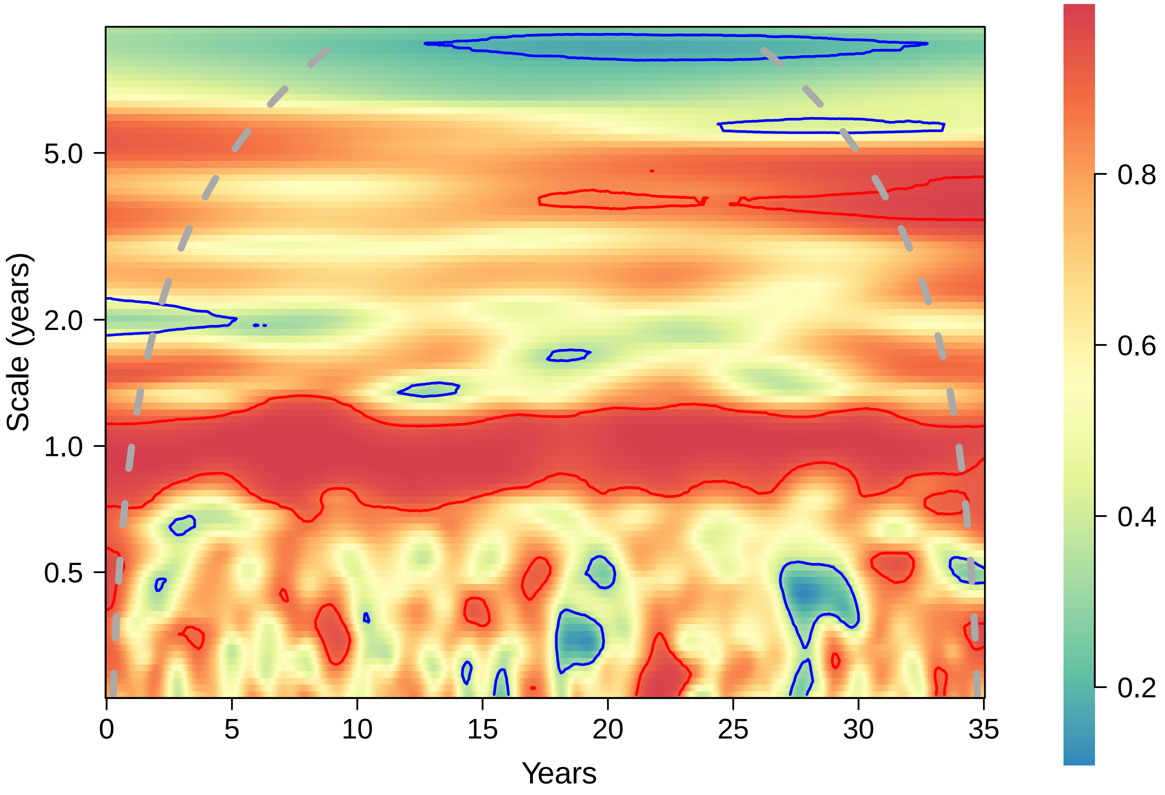

Figure S17: Wavelet modulus ratio in a 4-species community with compensation between the 2 most abundant species with a 9-year cycle. Blue and red lines respectively delineate regions of significantly lower and higher synchrony than the null model (independently fluctuating species, but conserving their original Fourier spectrum), at the 10% level. Significance is evaluated with 1000 surrogates.

We find signs of compensation, which is encouraging, but they are not significant throughout the whole time series, which implies that we might not detect long-term compensation as well as we would hope.

Appendix S8 - Effect of variation dominance on wavelet analyses

Highly skewed distribution of temporal variation among species

Skewed distributions of abundances can come not only from different average abundances (see Appendix S7), but also from a combination of the SAD with different amplitudes of temporal variation in abundances among species. A highly variable and abundant species, momentarily reaching higher numbers than all the rest of the species combined and whose numbers change more than the rest of the community, can drive the observed dynamics of the community up to the point that it possibly forbids compensation. This impossibility of compensation arises from the fact that under that scenario, no matter how the other species numbers combine, they may not be able to compensate the changes in numbers observed in the most abundant and variable species.

Starting from the simulation described in Appendix S7, in the case of compensation, we modeled a 4-species community in which we modified the distribution of abundances, so that the most abundant species had an amplitude 3 times higher than the sum of the amplitudes of the other species (ratio found within the wader group, at or below the annual time scale). To do so, we increased the amplitude of variation of the most abundant species while keeping the minimum constant, therefore modifying also its mean abundance, but not the distribution of abundances of the other species, i.e. for the most abundant species $\mu_{t,i} = \mu_{\min,i} + \kappa(\mu_{\max,i} - \mu_{\min,i})\left(1 + 0.5\sin\left(\frac{2\pi t}{P}\right)\right)$ where $\kappa = 7$ in a 4-species community.

In this case, we still found signs of compensation, but weakly and for longer time scales than the one assumed in the simulations (i.e., above 2 years). There was intermittent detection of compensation at short (monthly) time scales though, but this detection was not homogeneous throughout the time series, as it should have if compensation was detectable. This absence of detection is likely not an artefact of the method, but rather a limitation of the compensation concept. As we sketched above, whenever one species fluctuates much more than the rest, it is impossible for a change in numbers of the dominant species to be fully compensated by a change in numbers of the rest of the species.

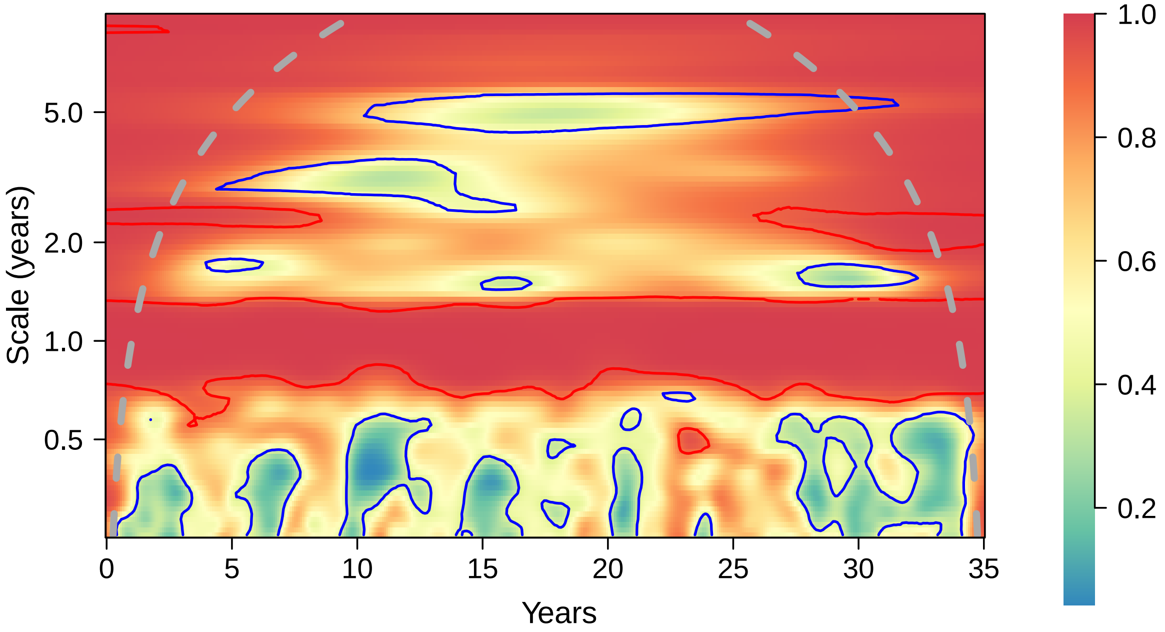

Figure S18: Wavelet modulus ratio, for 4 species with compensation, with 1 species having an amplitude of variation 3 times higher than the sum of the others. Blue and red lines respectively delineate regions of significantly lower and higher synchrony than the null model (independently fluctuating species, but conserving their original Fourier spectrum), at the 10% level.

Dynamics including species in antiphase

In the most extreme case of compensation, we analysed 2 species with opposed annual phases, under the constraint that one of them has a much higher amplitude of variation. We therefore explore the same issue of variation dominance in a different context. We mimicked this time the dynamics of the subset formed by the cormorant and the group heron/egret, in which compensation was not detected despite being apparent on time series through antiphase behaviour (Figs. 5 and 6 in the main text). The group heron/egret is indeed more abundant and has an amplitude of variation nearly 4 times higher than the cormorant.

At each time step, abundances are drawn from a lognormal distribution $\mathcal{LN}(\boldsymbol{\nu}_t, \boldsymbol{\Sigma})$ where $\nu_{t,i} = (\nu_{\max,i} - \nu_{\min,i})\left(0.5 + 0.5\sin(\frac{2\pi t}{P} + \phi_i)\right)$ with $\nu_{\max/\min,\text{i}}$ the maximum and minimum log amplitude taken directly from the observations, the phase $\boldsymbol{\phi} = (0, \pi)$ to force the antiphase in the dynamics, and $\Sigma_{ii} = 1$ and $\Sigma_{ij} = 0, \forall i \neq j$. This implies that we do not assume compensation to be present at lower or higher temporal scales than the year.

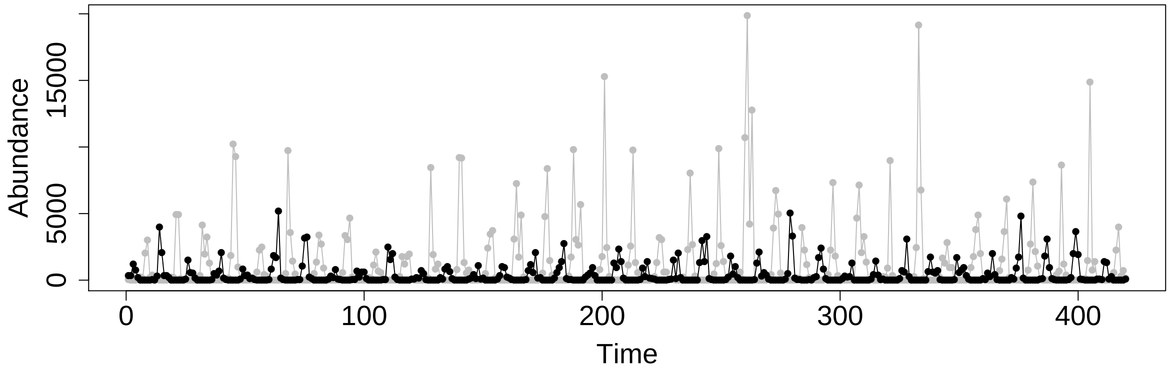

Figure S19: Simulated time series for 2 species in antiphase

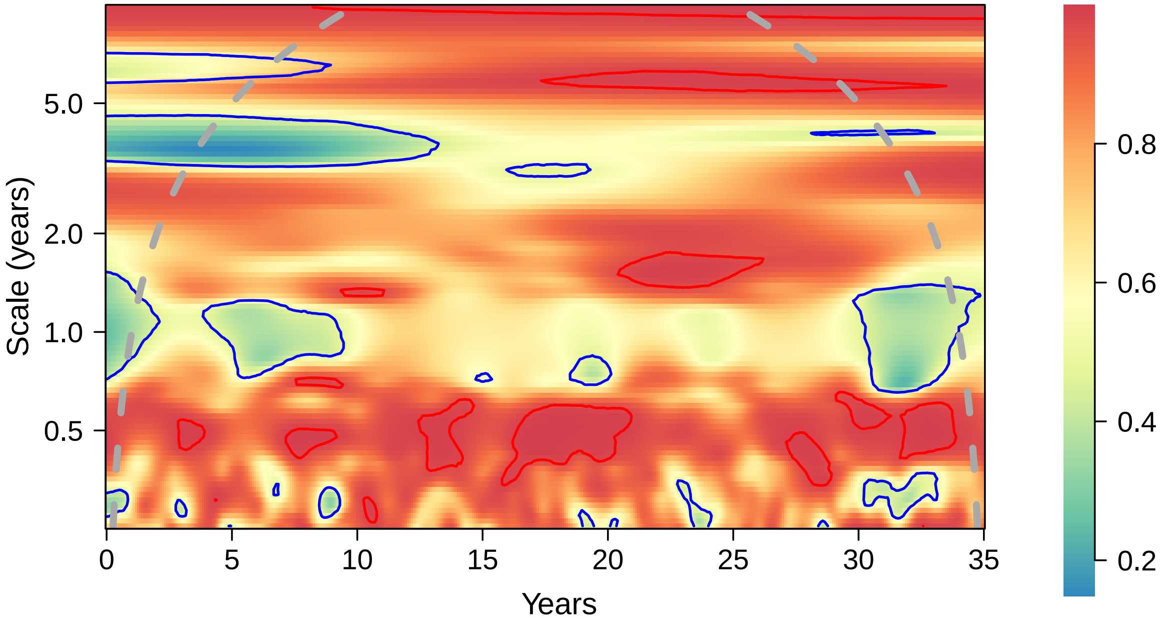

Figure S20: Wavelet modulus ratio for 2 species in antiphase, one of them having an amplitude of variation 4 times higher than the other one. Blue and red lines respectively delineate regions of significantly lower and higher synchrony than the null model (independently fluctuating species, but conserving their original Fourier spectrum), at the 10% level.

"Compensation" (species being in antiphase) is then poorly detected at the annual scale, the detection being as in the previous section intermittent instead of being constant. The randomness of peak heights also created some compensation at longer time scales in this simulation, but this should not be overinterpreted.

References

Gross, K., Cardinale, B.J., Fox, J.W., Gonzalez, A., Loreau, M., Wayne Polley, H., Reich, P.B. & van Ruijven, J. (2014) Species richness and the temporal stability of biomass production: a new analysis of recent biodiversity experiments. *The American Naturalist* **183**, 1–12.
